# Supplementary material for: RNA-binding proteins of KHDRBS and IGF2BP families control the oncogenic activity of MLL-AF4
Source: Nat Commun. 2022 Nov 5;13:6688. doi: 10.1038/s41467-022-34558-1 (PMC9637093; doi:10.1038/s41467-022-34558-1)
Supplement: Supplementary file 1 — Supplementary Information [file 41467_2022_34558_MOESM1_ESM.pdf]

RNA-Binding Proteins of KHDRBS and IGF2BP families control the Oncogenic Activity of MLL-AF4

Okuda *et al*.

SUPPLEMENTARY INFORMATION

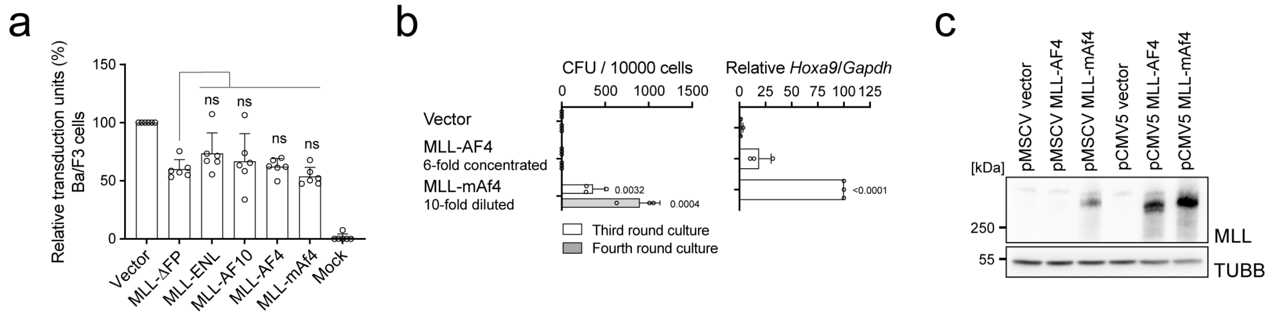

Supplementary Fig. 1. Virus production from the constructs of *MLL* fusion genes. Related to Fig. 1.

(a) Transduction of recombinant viruses into a murine pro-B cell line. Relative transduction units of retroviruses carrying various *MLL* fusion genes in murine Ba/F3 cells were determined using qPCR of the genomic DNA, as shown in Fig. 1f (n=6).

(b) Transforming ability of MLL-AF4 with an excess virus titer. Transforming ability using a 6-fold concentrated pool of MLL-AF4 was examined using the myeloid progenitor transformation assay, as shown in Fig. 1a, along with a 10-fold diluted pool of the MLL-mAf4 virus (n=3).

(c) Protein expression of MLL-AF4 and MLL-mAf4 driven by various promoters. Western blot analysis was performed on the whole cell lysates of 293T cells transfected with the expression vectors by which MLL fusions are transcribed from the LTR (pMSCV) or pCMV promoters (pCMV5).

Data are presented as the mean ± SD of indicated biologically independent replicates (a, b). P-value was calculated by one-way ANOVA followed by Tukey's test (a, b). Source data are provided as a Source Data file.

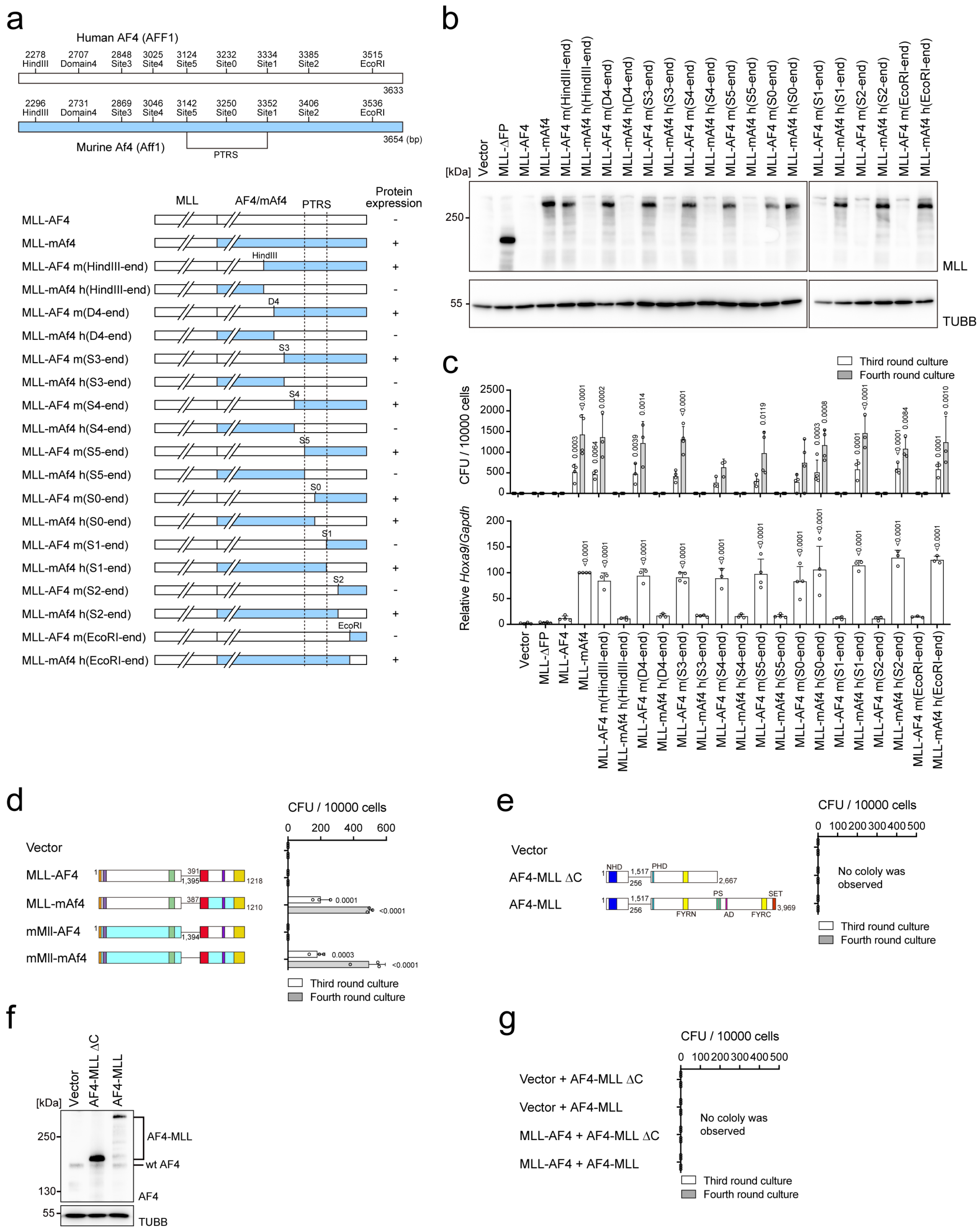

Supplementary Fig. 2. Post-transcriptional regulatory sequence affects AF4 protein expression. Related to Fig. 2.

(a) Schematic representation of various domain-swapping mutants of MLL-AF4 and MLL-mAf4. Domain swapping mutants are shown in blue (mouse) and white (human). The positions in the coding

sequences are indicated on top.

(b) Protein expression of the domain-swapping mutants of MLL-AF4 and MLL-mAf4. Western blot analysis was performed on the whole cell lysates of 293T cells transfected with the MLL fusion expression vectors, as shown in Fig. 1d.

(c) Transforming ability of the domain-swapping mutants of MLL-AF4 and MLL-mAf4. The MLL fusion constructs shown in Supplementary Fig. 1A were examined for transformation of HSPCs under an ex vivo myeloid culture condition, as shown in Fig. 1a (n=4: Vector, MLL-ΔFP, MLL-AF4, MLL-mAf4, MLL-AF4 m(S3-end), MLL-mAf4 h(S3-end), MLL-AF4 m(S5-end), MLL-mAf4 h(S5-end), MLL-AF4 m(S3-end), MLL-mAf4 h(S3-end); n=3: the others).

(d) Transforming ability of the domain-swapping mutants of murine/human MLL and murine/human AF4. Ex vivo myeloid progenitor transformation assays were performed as shown in Fig.1a (n=3).

(e) Transforming ability of AF4-MLL. Ex vivo myeloid progenitor transformation assays were performed as shown in Fig.1a (n=3).

(f) Protein expression of AF4-MLL. Western blot analysis was performed on the whole cell lysates of 293T cells transfected with AF4-MLL expression vectors, as shown in Fig. 1d.

(g) Transforming ability of MLL-AF4 in the presence of AF4-MLL. Two viruses were doubly transduced to HSPCs and their colony forming abilities were monitored as shown in Fig.1a (n=2).

Data are presented as the mean ± SD of indicated biologically independent replicates (c, d, e, g). P-value was calculated by one-way ANOVA followed by Tukey's test (c, d). Source data are provided as a Source Data file.

a

MLL-mAf4	Name	Day of sacrifice	CD3e	B220	CD11b	Spleen (g)
1		200	(-)	(-)	(+)	N.D.
2		239	(-)	(-)	(+)	0.3285
3	AML#1	159	(-)	(-)	(+)	0.5531
4		189	(-)	(-)	(+)	0.4235
5		132	(-)	(-)	(+)	0.3900
6	AML#2	132	(-)	(-)	(+)	0.6207
7		154	(-)	(-)	(+)	0.5070
8		154	(-)	(-)	(+)	0.8219
9		119	(-)	(-)	(+)	0.4207
10	AML#3	141	(-)	(-)	(+)	0.5083
11		202	(-)	(-)	(+)	0.5645

MLL-AF4 sPTRS	Name	Day of sacrifice	CD3e	B220	CD11b	Spleen (g)
1	AML#1	145	(-)	(-)	(+)	0.5137

b

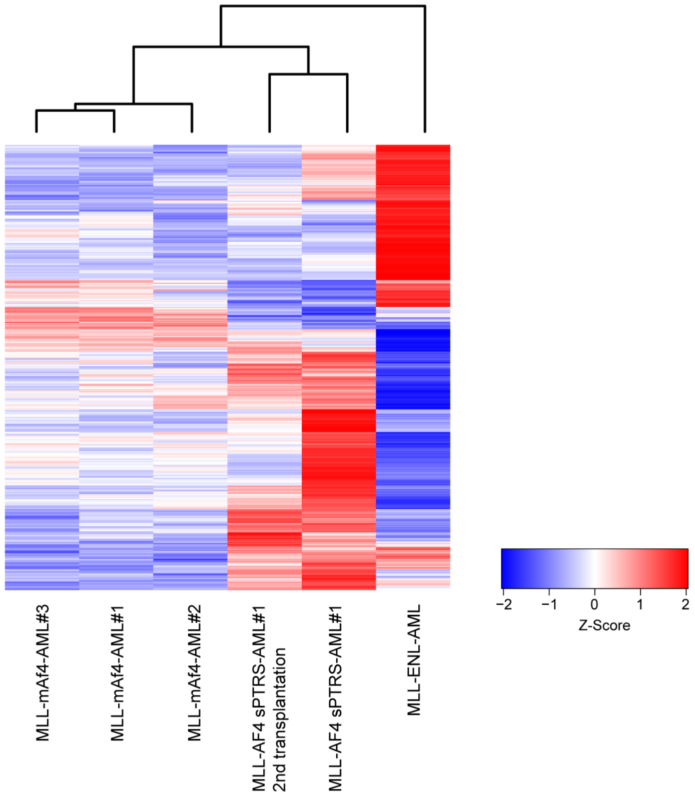

Supplementary Fig. 3. Immunophenotypes of leukemia induced by MLL-AF4 sPTRS. Related to Fig. 3.

(a) Surface marker expression of bone marrow cells from leukemic mice. Latency, antigen expression, and the weight of spleen of all leukemia cases are shown.

(b) Expression profiles of bone marrow cells harvested from MLL-AF4, MLL-mAf4 and MLL-ENL leukemic mice. Hierarchical clustering was performed by heatmap.2 function in r environment.

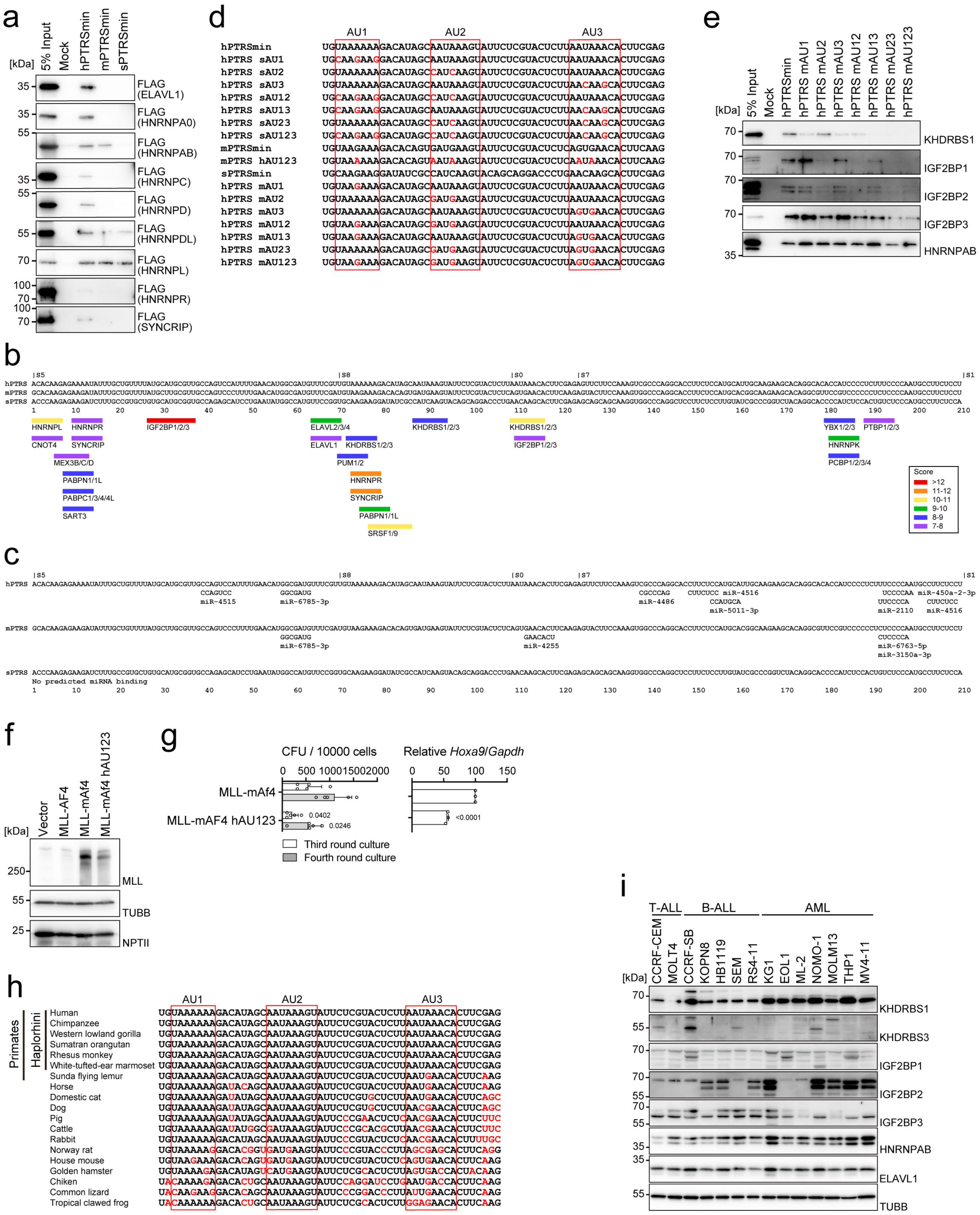

Supplementary Fig. 4. Three AU-rich sequences were the scaffold for association of various RBPs. Related to Fig. 4.

(a) Association of various RBPs with the PTRS. Immunoprecipitation-western blot analysis using the PTRS RNAs was performed on the lysates of 293T cells transiently expressing FLAG-tagged proteins

as shown in Fig. 4c.

(b) The RNA sites specifically recognized by various RBPs in the PTRS of human *AF4*. A result of CISBP-RNA database analysis is shown as in Fig. 4d for a broader region of the PTRS.

€ Predicted miRNA and lncRNA binding sites on the PTRSs. Results of miRbase and lncRNAdb database analyses are shown.

(d) Sequence of the mutated PTRSs. Mutations introduced in each mutant are highlighted in red.

(e) Association of endogenous RBPs to the PTRS of human *AF4* partially replaced by mouse sequences. Immunoprecipitation-western blot analysis using the PTRS RNAs shown in Fig. S4C was performed on the lysates of 293T cells, as shown in Fig. 4g. AU-rich sites of human PTRS were replaced by the corresponding mouse sequence.

(f) Protein expression of the MLL-mAF4 mutant with human AU-rich motifs in 293T cells. Protein level of the MLL-mAf4 mutant was analyzed as mentioned in Fig. 1d.

(g) Transforming ability of the MLL-mAf4 mutant, the AU-rich sites of which were replaced by human sequences. An MLL-mAf4 construct carrying human AU-rich sites was examined for transformation of HSPCs under ex vivo myeloid culture conditions, as shown in Fig. 1a (n=5). *Hoxa9* expression normalized to *Gapdh* is shown as the relative value of MLL-mAF4 (set to 100) (n=3). Data are presented as the mean ± SD of indicated biologically independent replicates. P-value was calculated by two tailed T-test.

(h) Sequence alignment of the PTRS of various organisms. AU-rich sites are highlighted by red rectangles. Bases different from human AU-rich sites are shown in red letters.

(i) Expression of RNA-binding proteins in various leukemia cell lines. Western blotting of RBPs was performed using whole cell lysates of leukemia cell lines of various lineages.

Source data are provided as a Source Data file.

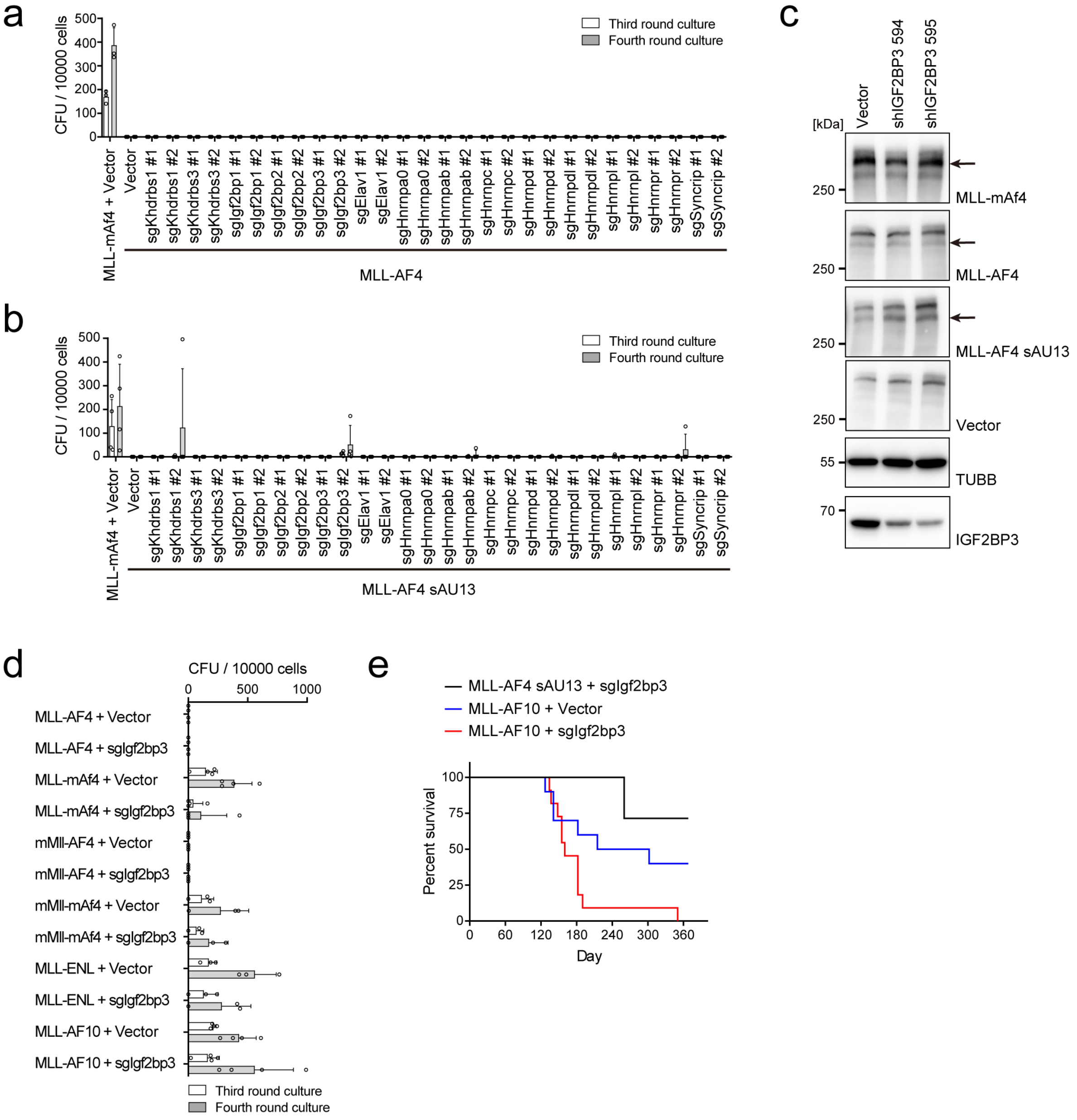

Supplementary Fig. 5. sgRNA screening of the transformation capabilities of the MLL-AF4 sAU13 mutant and protein expression of the mutant after depletion of IGF2BP3.

(a) Screening of RBPs responsible for post-transcriptional inactivation of MLL-AF4. The MLL-AF4 construct was examined for transformation of HSPCs under an ex vivo myeloid culture condition with co-transduction of various knockout constructs for RBPs, as shown in Fig. 5a (n=3).

(b) Screening of RBPs responsible for post-transcriptional inactivation of MLL-AF4 sAU13. The MLL-AF4 construct carrying synonymous mutations at AU1 and 3 was examined for transformation of HSPCs under an ex vivo myeloid culture condition with co-transduction of various knockout constructs for RBPs, as shown in Fig. 5a. Data are shown as the mean ± SD of three biological replicates (a, b) (n=3: MLL-AF4 sAU13 + sgHnrnpa0#1, MLL-AF4 sAU13 + sgHnrnpa0#2, MLL-AF4 sAU13 + sgHnrnpc#1, MLL-AF4 sAU13 + sgHnrnpc#2, MLL-AF4 sAU13 + sgHnrnpdl#1,

MLL-AF4 sAU13 + sgHnrnpdl#2, MLL-AF4 sAU13 + sgHnrnpl#1, MLL-AF4 sAU13 + sgHnrnpl#2, MLL-AF4 sAU13 + sgSyncrip#1, MLL-AF4 sAU13 + sgSyncrip#2; n=4: the others).

(c) Effects of knocking down *IGF2BP3* on protein expression of MLL-AF4. 293T cells were transduced with two different sh-RNA constructs for *IGF2BP3* and subsequently transfected with various MLL-AF4 expression vectors. Western blot analysis was performed on the whole cell lysate of transfected cells using anti-MLL antibody. Western blot analysis of TUBB and IGF2BP3 was also performed.

(d) Effects of Igf2bp3 knockout on various MLL fusion mediated transformation. MLL fusion constructs were doubly transduced with the CRISPR/Cas9 knockout construct for *Igf2bp3* into murine HSPCs and examined for their transforming abilities under ex vivo myeloid culture conditions, as shown in Fig. 5a (n=4: MLL-mAf4 + Vector, MLL-mAf4 + sgIgf2bp3, MLL-AF10 + Vector, MLL-AF10 + sgIgf2bp3; n=3: mMll-AF4 + Vector, mMll-AF4 + sgIgf2bp3, mMll-mAf4 + Vector, mMll-mAf4 + sgIgf2bp3, MLL-ENL + Vector, MLL-ENL + sgIgf2bp3; n=2: MLL-AF4 + Vector, MLL-AF4 + sgIgf2bp3).

(e) Leukemogenic potential of MLL fusion-immortalized cells with or without Igf2bp3 knockout in vivo. MLL-AF10-immortalized cells with or without Igf2bp3 knockout and MLL-AF4 sAU13/Igf2bp3 knockout-immortalized cells established in Fig. 5a were transplanted into syngeneic mice. (MLL-AF4 sAU13 sgIgf2bp3, n = 7; MLL-AF10 vector, n = 10; MLL-AF10 sgIgf2bp3, n = 11). As for the transplantation experiment of MLL-AF4 sAU13/Igf2bp3 knockout-immortalized cells, two recipient mice died of unknown causes. Three recipient mice became sick with B-ALL, but the transgene expression was not detected by RT-PCR, therefore excluded as spontaneous leukemia cases. p-value (p=0.0377) was calculated by log-rank test (MLL-AF10 vector vs. MLL-AF10 sgIgf2bp3).

Data are presented as the mean ± SD of indicated biologically independent replicates (a, b, d). Source data are provided as a Source Data file.

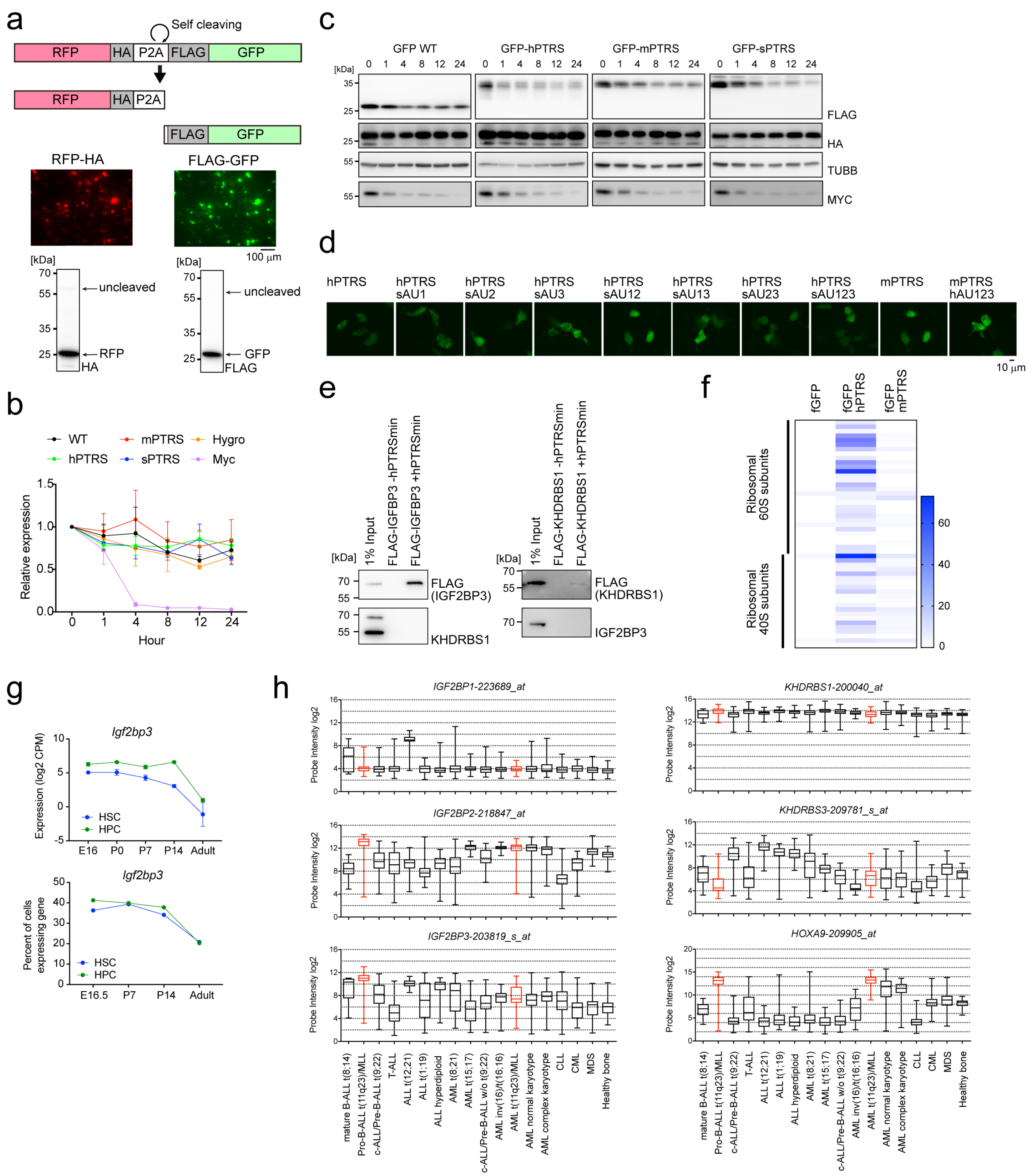

Supplementary Fig. 6. Fluorescent reporter recapitulated post-transcriptional regulation of MLL-AF4 expression. Related to Fig. 6.

(a) Schematic representation of the self-cleaving GFP/RFP reporter system. Cleaving efficiency of the P2A sequence of the fluorescent reporter. 293T cells transfected with the reporter plasmid were analyzed using fluorescence microscopy and western blotting.

(b) mRNA stability of the reporter genes. The reporter plasmids were transfected into 293T cells and treated with actinomycin D for 24 h. The mRNAs were purified and quantified by RT-qPCR. The

expression level was normalized by that of GAPDH. MYC gene was used as a positive control. Data are shown as the mean ± SD of three biological replicates.

(c) Protein stability of the reporter proteins. The reporter plasmids were transfected into 293T cells and treated with cycloheximide for 24 h. The protein levels were visualized by western blotting. MYC protein was used as a positive control.

(d) Subcellular localization of fluorescence signals of various GFP-tagged PTRS reporters.

(e) Non-simultaneous binding of RBPs to hPTRS. Two-step IP analysis of IGF2BP3 and KHDRBS1 on the hPTRS RNA oligonucleotide was performed. The FLAG-tagged RBPs were transfected into 293T cells. Then the lysate was mixed with the hPTRS RNA oligonucleotide and subjected to subsequent pull down of FLAG-tagged proteins and the bait RNAs.

(f) Associated factors for the GFP-PTRS reporter proteins. Heatmap representation of the scores of the ribosomal proteins associated with PTRS reporters.

(g) scRNA-seq data of the murine embryonic HSPCs in the public database. The expression levels and percentages of the cells expressing Igf2bp3 during developmental stages were plotted. CPM: count per million, HSC: hematopoietic stem cell, HPC: hematopoietic progenitor cell. (Li et al., 2020[18], GSE128761)

(h) Expression of RBPs in the human leukemia specimens. The log2 probe intensities in various subtypes of the leukemias were plotted (MILE stage 2[19], GSE13164). Data are shown as the boxplot. The center line indicates the median value. The box limits indicate the first and third quartiles. The bar limits indicate the maximum and minimum values. (mature B-ALL t(8;14): n=13, Pro-B-ALL t(11q23)/MLL: n=70, c-ALL/Pre-B-ALL t(9;22): n=122, T-ALL: n=174, ALL t(12;21): n=58, ALL t(1;19): n=36, ALL hyperdiploid: n=40, c-ALL/Pre-B-ALL w/o t(9;22): n=237, AML t(8;21): n=40, AML t(15;17): n=37, AML inv(16)/t(16;16): n=28, AML t(11q23)/MLL: n=38, AML normal karyotype: n=351, AML complex karyotype: n=48, CLL: n=448, CML: n=76, MDS: 206, Healthy bone: n=74) ALL: acute lymphoblastic leukemia, AML: acute myeloid leukemia, CLL: chronic lymphocytic leukemia, CML: chronic myeloid leukemia, MDS: myelodysplastic syndromes.

Source data are provided as a Source Data file.

Supplementary Table 1, Materials

REAGENT or RESOURCE	SOURCE	IDENTIFIER
Antibodies		
Mouse monoclonal anti-FLAG M2	Sigma-Aldrich	Cat#F3165 RRID: AB_259529
Rabbit polyclonal anti-FLAG	Sigma-Aldrich	Cat#F7425 RRID: AB_439687
Rat monoclonal anti-HA (clone 3F10)	Roche	Cat#11867423001 RRID: AB_390919
Rabbit monoclonal anti-MLL (clone D2M7U)	Cell Signaling Technology	Cat#14689 RRID: AB_2688009
Rabbit polyclonal anti-TUBB2C	Invitrogen	Cat#PA5-25050 RRID: AB_2542550
Rabbit polyclonal anti-neomycin phosphotransferase II	Merck-Millipore	Cat#06-747 RRID: AB_310234
Rabbit polyclonal anti-KHDRBS1	Bethyl Laboratories	Cat#A302-110A RRID: AB_1604287
Rabbit polyclonal anti-KHDRBS1	Bethyl Laboratories	Cat#A302-111A RRID: AB_1604288
Rabbit polyclonal anti-KHDRBS3	Bethyl Laboratories	Cat#A303-192A RRID: AB_10951667
Rabbit polyclonal anti-IGF2BP1	Bethyl Laboratories	Cat#A303-423A RRID: AB_10954093
Rabbit polyclonal anti-IGF2BP1	Bethyl Laboratories	Cat#A303-424A RRID: AB_10951834

Rabbit polyclonal anti-IGF2BP2	Bethyl Laboratories	Cat#A303-317A RRID: AB_10952227
Rabbit polyclonal anti-IGF2BP3	Bethyl Laboratories	Cat#A303-425A RRID: AB_10953339
Rabbit polyclonal anti-IGF2BP3	Bethyl Laboratories	Cat#A303-426A RRID: AB_10951696
Mouse monoclonal anti-HNRNPAB (clone G10)	Santa Cruz Biotechnology	Cat#sc-376411 RRID: AB_11150309
Rabbit monoclonal anti-RPL5	Cell Signaling Technology	Cat#51345 RRID: AB_2799391
Rabbit polyclonal anti-RPL7a	Cell Signaling Technology	Cat#2415 RRID: AB_2182059
Rabbit polyclonal anti-RPL7	Bethyl Laboratories	Cat#A300-741A RRID: AB_2301241
Rabbit polyclonal anti-RPS14	Bethyl Laboratories	Cat#A304-031A RRID: AB_2621280
Rabbit polyclonal anti-AF4	Bethyl Laboratories	Cat#A302-344A RRID: AB_1850255
Rabbit monoclonal anti-MYC	Cell Signaling Technology	Cat#13987 RRID: AB_2631168
Rat monoclonal anti-human/mouse CD45R(B220) (clone RA3-6B2)-FITC	eBioscience	Cat#11-0452-82 RRID: AB_465054
Rat monoclonal anti-human/mouse CD45R(B220) (clone RA3-6B2)-PE	BioLegend	Cat#103208 RRID: AB_312993

Armenian hamster monoclonal anti-mouse CD3e (clone 145-2C11)-APC	eBioscience	Cat#17-0031-82 RRID: AB_469315
Armenian hamster monoclonal anti-mouse CD3e (clone 145-2C11)-APC	BioLegend	Cat#100312 RRID: AB_312677
Rat monoclonal anti-mouse CD11b (clone M1/70)-PE-Cyanine7	eBioscience	Cat#25-0112-82 RRID: AB_469588
Rat monoclonal anti-mouse CD11b (clone M1/70)-Brilliant Violet 510	BioLegend	Cat#101263 RRID: AB_2629529
Rat monoclonal anti-mouse Gr1 (clone RB6-8C5)-PE-Cyanine7	BioLegend	Cat#25-5931-82 RRID: AB_469663
Rat monoclonal anti-mouse Gr1 (clone RB6-8C5)-Brilliant Violet 421	BD Biosciences	Cat#562709 RRID: AB_2737736
Chemicals, Peptides, and Recombinant Proteins		
Recombinant murine stem cell factor	PeproTech	Cat#250-03
Recombinant murine interleukin-3	PeproTech	Cat#213-13
Recombinant murine granulocyte-macrophage colony-stimulating factor	PeproTech	Cat#315-03
Recombinant murine interleukin-6	PeproTech	Cat#216-16
Recombinant murine interleukin-7	PeproTech	Cat#217-17
Recombinant murine Flt3-ligand	PeproTech	Cat#250-31L
RetroNectin (Recombinant human fibronectin fragment)	Takara Bio	Cat#T100A
Lipofectamine 2000 transfection reagent	Thermo Fisher	Cat#11668027
Dynabeads M-280 streptavidin	Invitrogen	Cat#11205D
Anti-FLAG M2 magnetic beads affinity isolated antibody	Sigma-Aldrich	Cat#M8823
RapiGest SF	Waters	Cat#186001861
RNaseOUT recombinant ribonuclease inhibitor	Invitrogen	Cat#10777019
Micrococcal nuclease	Sigma-Aldrich	Cat#N3755-200U
Hoechst 33342 solution	Thermo Fisher Scientific	Cat#H3570
Protease inhibitor cocktail	Roche	Cat#11873580001

Actinomycin D	Wako Chemicals	Cat#018-21264
Cycloheximide	Nacalai Tesque	Cat#06741-91
Critical Commercial Assays		
RNeasy mini kit	Qiagen	Cat#74016
RNase-free DNase kit	Qiagen	Cat#79254
SuperScript III first-strand cDNA synthesis system	Invitrogen	Cat#18180051
High pure PCR template preparation kit	Roche	Cat#11796828001
c-Kit magnetic beads	Miltenyi Biotec	Cat#130-091-224
SureSelect strand specific RNA library prep kit	Agilent Technologies	Cat#G9691A
NextSeq 500/550 high output kit v2.5	Illumina	Cat#20024906
RNA6000 pico kit	Agilent Technologies	Cat#5067-1513
High sensitivity D1000 kit	Agilent Technologies	Cat#5067- 5585
Kapa library quantification kit	Kapa Biosystems	Cat#07960140001
Gibson assembly master mix	New England Biolabs	Cat#E26115
NucleoSpin RNA	Takara Bio	Cat#740955
PrimeScript RT reagent kit (Perfect real time)	Takara Bio	Cat#RR037B
Thunderbird SYBR qPCR mix	Takara Bio	Cat#QPS-201
Deposited Data		
Data files for RNA-seq	This paper	GSE201503
Experimental Models: Cell Lines		
Human: 293T cells	ATCC	CRL-3216 RRID: CVCL_0063
Human: PLAT-E cells	Morita et al., 2000[1]	N/A
Human: 293TN Producer cells	System Bioscience	LV900A-1 RRID: CVCL_UL49
Human: CCRF-CEM cells	JCRB Cell Bank	JCRB0033 RRID: CVCL_0207

Human: MOLT-4 cells	JCRB Cell Bank	JCRB9031 RRID: CVCL_0013
Human: CCRF-SB cells	JCRB Cell Bank	JCRB0032 RRID: CVCL_1860
Human: KOPN-8 cells	DSMZ	ACC552 RRID: CVCL_1866
Human: HB1119 cells	Tkachuk et al., 1992[2]	RRID: CVCL_8227
Human: SEM cells	DSMZ	ACC546 RRID: CVCL_0095
Human: RS4;11 cells	ATCC	CRL-1873 RRID: CVCL_0093
Human: KG-1 cells	JCRB Cell Bank	JCRB0065 RRID: CVCL_0374
Human: EOL-1 cells	Obtained from Michael Cleary	RRID: CVCL_0258
Human: ML-2 cells	DSMZ	ACC15 RRID: CVCL_1418
Human: NOMO-1 cells	JCRB Cell Bank	IFO50474 RRID: CVCL_1609
Human: MOLM-13 cells	DSMZ	ACC554 RRID: CVCL_2119
Human: THP-1 cells	ATCC	TIB-202 RRID: CVCL_0006

Human: MV4-11 cells	ATCC	CRL-9591<br>RRID:<br>CVCL_0064
Mouse: MS-5 cells	DSMZ	ACC441<br>RRID:<br>CVCL_2128
Mouse: MS-5-neo cells	This paper	N/A
Mouse: Ba/F3 cells	Obtained from RIKEN BRC	
Mouse: MEF clone C2-20 cells	Okuda et al., 2015[3]	
Experimental Models: Organisms/Strains		
Mouse: C57BL/6JJcl	CLEA Japan	N/A
Oligonucleotides		
TaqMan Probe, mouse *Hoxa9*	Life Technologies	Cat#Mm00439364_m1
TaqMan Probe, mouse *Gapdh*	Life Technologies	Cat#Mm99999915_g1
TaqMan Probe, mouse *Kit*	Life Technologies	Cat#Mm00445212_m1
TaqMan Probe, mouse *Meis1*	Life Technologies	Cat#Mm00487664_m1
TaqMan Probe, mouse *Hoxa6*	Life Technologies	Cat#Mm00550244_m1
TaqMan Probe, mouse *Hoxa7*	Life Technologies	Cat#Mm00657963_m1
TaqMan Probe, mouse *Hoxa10*	Life Technologies	Cat#Mm00433966_m1
TaqMan Probe, mouse *Hoxa11*	Life Technologies	Cat#Mm00439360_m1
TaqMan Probe, mouse *Myc*	Life Technologies	Cat#Mm00487804_m1
TaqMan Probe, human *GAPDH*	Life Technologies	Cat#Hs02786624_g1

TaqMan Probe, human *AF4*	Life Technologies	Cat#Hs01014712_m1
Oligonucleotides for gene editing and shRNA, see Supplementary Table 2	This paper	N/A
Custom TaqMan Probes, see Supplementary Table 3	Life Technologies	N/A
Primers for SYBR green qPCR, see Supplementary Table 4	This paper	N/A
Biotinylated-RNA oligo, see Supplementary Figure 4C	Thermo Fisher Scientific	N/A
Recombinant DNA		
pMDLg/pRRE	Dull et al., 1998[4]	Addgene: 12251 RRID: Addgene_12251
pRSV-Rev	Dull et al., 1998[4]	Addgene: 12253 RRID: Addgene_12253
pMD2.G	Gift from Didier Trono	Addgene: 12259 RRID: Addgene_12259
pMSCV-neo	Clontech Laboratories	Cat#634401
pMSCV-neo-MLL(5')	Okuda et al., 2015[3]	
pMSCV-neo-MLL-ENL	Yokoyama et al., 2008[5]	
pMSCV-neo-MLL-AF10	DiMartino et al., 2002[6]	
pMSCV-neo-MLL-AF4	This paper	N/A
pMSCV-neo-MLL-mAf4	This paper	N/A
pMSCV-neo-MLL-AF4 m(HindIII-end)	This paper	N/A
pMSCV-neo-MLL-AF4 m(D4-end)	This paper	N/A
pMSCV-neo-MLL-AF4 m(S3-end)	This paper	N/A
pMSCV-neo-MLL-AF4 m(S4-end)	This paper	N/A
pMSCV-neo-MLL-AF4 m(S5-end)	This paper	N/A
pMSCV-neo-MLL-AF4 m(S0-end)	This paper	N/A

pMSCV-neo-MLL-AF4 m(S1-end)	This paper	N/A
pMSCV-neo-MLL-AF4 m(S2-end)	This paper	N/A
pMSCV-neo-MLL-AF4 m(EcoRI-end)	This paper	N/A
pMSCV-neo-MLL-AF4 mPTRS	This paper	N/A
pMSCV-neo-MLL-AF4 sPTRS	This paper	N/A
pMSCV-neo-MLL-AF4 s(S5-S8)	This paper	N/A
pMSCV-neo-MLL-AF4 s(S8-S7)	This paper	N/A
pMSCV-neo-MLL-AF4 s(S7-S1)	This paper	N/A
pMSCV-neo-MLL-AF4 sAU1	This paper	N/A
pMSCV-neo-MLL-AF4 sAU2	This paper	N/A
pMSCV-neo-MLL-AF4 sAU3	This paper	N/A
pMSCV-neo-MLL-AF4 sAU12	This paper	N/A
pMSCV-neo-MLL-AF4 sAU13	This paper	N/A
pMSCV-neo-MLL-AF4 sAU23	This paper	N/A
pMSCV-neo-MLL-AF4 sAU123	This paper	N/A
pMSCV-neo-MLL-mAf4 h(HindIII-end)	This paper	N/A
pMSCV-neo-MLL-mAf4 h(D4-end)	This paper	N/A
pMSCV-neo-MLL-mAf4 h(S3-end)	This paper	N/A
pMSCV-neo-MLL-mAf4 h(S4-end)	This paper	N/A
pMSCV-neo-MLL-mAf4 h(S5-end)	This paper	N/A
pMSCV-neo-MLL-mAf4 h(S0-end)	This paper	N/A
pMSCV-neo-MLL-mAf4 h(S1-end)	This paper	N/A
pMSCV-neo-MLL-mAf4 h(S2-end)	This paper	N/A
pMSCV-neo-MLL-mAf4 h(EcoRI-end)	This paper	N/A
pMSCV-neo-MLL-mAf4 hPTRS	This paper	N/A
pMSCV-neo-MLL-mAf4 hAU123	This paper	N/A
pMSCV-hygro-AF4-MLL	This paper	N/A
pMSCV-hygro-AF4-MLL ΔC	This paper	N/A
pCDH-MSCV-MCS-EF1-Puro	System Biosciences	Cat#CD710B-1
pCDH-MSCV-MCS-EF1-Hygro	This Paper	N/A
pCDH-MSCV-RFP-HA-P2A-FLAG-GFP-EF1-Hygro	This Paper	N/A
pCDH-MSCV-RFP-HA-P2A-FLAG-GFP-hPTRS-EF1-Hygro	This Paper	N/A

pCDH-MSCV-RFP-HA-P2A-FLAG-GFP-mPTRS-EF1-Hygro	This Paper	N/A
pCDH-MSCV-RFP-HA-P2A-FLAG-GFP-sPTRS-EF1-Hygro	This Paper	N/A
pCDH-MSCV-RFP-HA-P2A-FLAG-GFP-hPTRS-sAU1-EF1-Hygro	This Paper	N/A
pCDH-MSCV-RFP-HA-P2A-FLAG-GFP-hPTRS-sAU2-EF1-Hygro	This Paper	N/A
pCDH-MSCV-RFP-HA-P2A-FLAG-GFP-hPTRS-sAU3-EF1-Hygro	This Paper	N/A
pCDH-MSCV-RFP-HA-P2A-FLAG-GFP-hPTRS-sAU12-EF1-Hygro	This Paper	N/A
pCDH-MSCV-RFP-HA-P2A-FLAG-GFP-hPTRS-sAU13-EF1-Hygro	This Paper	N/A
pCDH-MSCV-RFP-HA-P2A-FLAG-GFP-hPTRS-sAU23-EF1-Hygro	This Paper	N/A
pCDH-MSCV-RFP-HA-P2A-FLAG-GFP-hPTRS-sAU123-EF1-Hygro	This Paper	N/A
pCDH-MSCV-RFP-HA-P2A-FLAG-GFP-STOP-hPTRS-EF1-Hygro	This Paper	N/A
pCDH-MSCV-RFP-HA-P2A-FLAG-GFP-mPTRS-hAU123-EF1-Hygro	This Paper	N/A
pCDH-MSCV-RFP-HA-P2A-FLAG-GFP-Rx12-EF1-Hygro	This Paper	N/A
pCDH-MSCV-FLAG-GFP-EF1-Hygro	This Paper	N/A
pCDH-MSCV-mCherry-KHDRBS1-EF1-Puro	This Paper	N/A
pCDH-MSCV-mCherry-KHDRBS3-EF1-Puro	This Paper	N/A
pCDH-MSCV-mCherry-IGF2BP1-EF1-Puro	This Paper	N/A
pCDH-MSCV-mCherry-IGF2BP2-EF1-Puro	This Paper	N/A
pCDH-MSCV-mCherry-IGF2BP3-EF1-Puro	This Paper	N/A
pCDH-MSCV-mCherry-HNRNPAB-EF1-Puro	This Paper	N/A
pCDH-MSCV-MCS-EF1-copGFP	System Biosciences	Cat#CD711B-1
pCDH-MSCV-IGF2BP3-EF1-copGFP	This Paper	N/A
pCMV5-FLAG-KHDRBS1	This Paper	N/A

pCMV5-FLAG-KHDRBS3	This Paper	N/A
pCMV5-FLAG-IGF2BP1	This Paper	N/A
pCMV5-FLAG-IGF2BP2	This Paper	N/A
pCMV5-FLAG-IGF2BP3	This Paper	N/A
pCMV5-FLAG-ELAVL1	This Paper	N/A
pCMV5-FLAG-HNRNPA0	This Paper	N/A
pCMV5-FLAG-HNRNPAB	This Paper	N/A
pCMV5-FLAG-HNRNPC	This Paper	N/A
pCMV5-FLAG-HNRNPD	This Paper	N/A
pCMV5-FLAG-HNRNPDL	This Paper	N/A
pCMV5-FLAG-HNRNPL	This Paper	N/A
pCMV5-FLAG-HNRNPR	This Paper	N/A
pCMV5-FLAG-SYNCRIP	This Paper	N/A
pKLV2-EF1a-Cas9Bsd-W	Tzelepis et al., 2016[7]	Addgene: 68343 RRID: Addgene_68343
pKLV2-U6gRNA5(BbsI)-PGKpuro2ABFP-W	Tzelepis et al., 2016[7]	Addgene: 67974 RRID: Addgene_67974
pX330-U6-Chimeric_BB-CBh-hSpCas9	Cong et al., 2013[8]	Addgene: 42230 RRID: Addgene_42230
pX335-U6-Chimeric_BB-CBh-hSpCas9n(D10A)	Cong et al., 2013[8]	Addgene: 42235 RRID: Addgene_42335
Software and Algorithms		
GraphPad Prism 9.3.1	GraphPad Software	https://www.graphpad.com/scientific-software/prism/
Xcalibur	Thermo Fisher Scientific	Cat#OPTON-30965
CHOPCHOP	Labun et al., 2019[9]	http://chopchop.cbu.uib.no/

CISBP-RNA	Ray et al., 2013[10]	http://cisbp-rna.ccbr.utoronto.ca/index.php
miRBase	Kozomara et al., 2019[11]	https://mirbase.org/
lncRNAdb	Amaral et al., 2011[12]	http://lncrnadb.org/
ImageJ	Schneider et al., 2012[13]	https://imagej.nih.gov/ij/
FlowJo v10.8.1	BD Biosciences	https://www.flowjo.com/
Bcl2fastq ver.2.20.0	Illumia	https://jp.support.illumina.com/downloads/bcl2fastq-conversion-software-v2-20.html
Star ver.2.7.7a	Dobin et al., 2013[14]	https://github.com/alexdobin/STAR/
Rsem ver.1.3.3	Li and Dewey, 2011[15]	https://github.com/deweylab/RSEM
R ver.4.1.2	R Core Team	https://www.r-project.org/
R studio ver.2021.09.1	RStudio Team	https://www.rstudio.com/
edgeR ver.3.34.1	Robinson et al. 2010[16]	https://bioconductor.org/packages/edgeR/
Seurat ver.4.1.0	Satija Lab[17]	https://satijalab.org/seurat
Source of Public Data Analysis		
Murine fetal HSPCs scRNA-seq data	Li et al., 2020[18]	GSE128761
Human leukemia microarray data	MILE stage 2[19]	GSE13164

Supplementary Table 2. Oligonucleotides for gene editing and shRNA-mediated knockdown.

Target name	Target sequence
sgKhdrbs1#1	GAACCCACCACCGTCGCGTC
sgKhdrbs1#2	CGGGTCGAGCGAGTCCTTCT
sgKhdrbs3#1	AACGGCCGCCGAGCTCACCT
sgKhdrbs3#2	CGCTTCAGGGAATTGCCACG
sgIgf2bp1#1	TCATCGCCCAGTGCTCGTCG
sgIgf2bp1#2	ATAGCGCCTACATACTGCGT
sgIgf2bp2#1	CGCGCCATCGAGACCCTCTC
sgIgf2bp2#2	AGGTCGTCGGCGGTGACGGC
sgIgf2bp3#1	ACGCGTAGCCCGTCTTCACC
sgIgf2bp3#2	CTACGCGTTCGTGGACTGCC
sgElavl1#1	CTTATTCGGGATAAAGTAGC
sgElavl1#2	CGAAGTCTGTTCAGCAGCAT
sgHnrnpa0#1	TGTCGCGGGAGGATTCGGCG
sgHnrnpa0#2	TACGGGCCGATGAAGAGCGG
sgHnrnpab#1	GGCCTCGTGTCCGTTCTCGG
sgHnrnpab#2	GGACCACGACCGCACCGAGC
sgHnrnpc#1	ACACGGGAATTCATGGACCG
sgHnrnpc#2	GGACCGAGGATCTGTCTTGT
sgHnrnpd#1	GTCGGAGGAGCAGTTCGGAG
sgHnrnpd#2	GTTCTTACTGGCGTCGATCT
sgHnrnpdl#1	TAGCCGCCCCGCCCGCCAAT
sgHnrnpdl#2	GGAACAATGGCGGCGGCACA
sgHnrnpl#1	CTATTACGGCGGCGGCAACG
sgHnrnpl#2	GGCGGTGGTCGCTATTACGG
sgHnrnpr#1	TGCTTCATCGGGCCCTTTCG
sgHnrnpr#2	GCCCGATGAAGCAAAAATCA
sgSyncrip#1	TTTACTAGAGTCTGCTACTT
sgSyncrip#2	AAAGTAGCAGACTCTAGTAA
shIGF2BP3#1	TCTGCGGCTTGTAAGTCTATT
shIGF2BP3#2	TGTTGTAGTCTCACAGTATAA

Supplementary Table 3. Sequences of the custom TaqMan Probes.

Primer/probe name	Sequence
MSCV-EPS-Forward	TTGTCTCTGTCTGACTGTGTTTCTG
MSCV-EPS-Probe	CTGGCCCTAATTTTCA
MSCV-EPS-Reverse	GGTCAAACTTAAGGGAGTGGTAACA
Human MLL-Forward	CCAAGTTTGGTGGTCGCAATATAAA
Human MLL-Probe	CAGTGCTGCAAGATGAG
Human MLL-Reverse	CATCCATTGTAGATTCTGACATTTT
Mouse Gapdh locus-Forward	CCTCCTCCAATTCAACCCTTAAGAG
Mouse Gapdh locus-Probe	ATGCTGCCCTTACCCC
Mouse Gapdh locus-Reverse	TCTCCTGAGTTTACCTGATGAACCT

Supplementary Table 4. Sequences of the primers for SYBR green qPCR.

Primer name	Sequence
GAPDH-Forward	GAAATCCCATCACCATCTTCCAGG
GAPDH-Reverse	GAGCCCCAGCCTTCTCCATG
PTRS-Reporter-Forward	AAGCTGTACATGGAGGGCACC
PTRS-Reporter-Reverse	ATCACAGGGCCGTTGGATGG
Hygro-Forward	CTGATGCTTTGGGCCGAGGA
Hygro-Reverse	CGGAGCATATACGCCCGGAG
MYC -Forward	TAGTGGAAAACCAGCAGCCT
MYC -Reverse	TTCTCCTCCTCGTCGCAGTA

Supplemental references

1. Morita, S., Kojima, T. & Kitamura, T. Plat-E: an efficient and stable system for transient packaging of retroviruses. *Gene Ther* **7**, 1063-1066 (2000).

2. Tkachuk, D.C., Kohler, S. & Cleary, M.L. Involvement of a homolog of Drosophila trithorax by 11q23 chromosomal translocations in acute leukemias. *Cell* **71**, 691-700 (1992).

3. Okuda, H., Kanai, A., Ito, S., Matsui, H. & Yokoyama, A. AF4 uses the SL1 components of RNAP1 machinery to initiate MLL fusion- and AEP-dependent transcription. *Nat Commun* **6**,

8869 (2015).

4. Dull, T. *et al.* A third-generation lentivirus vector with a conditional packaging system. *J Virol* **72**, 8463-8471 (1998).

5. Yokoyama, A. & Cleary, M.L. Menin critically links MLL proteins with LEDGF on cancer-associated target genes. *Cancer cell* **14**, 36-46 (2008).

6. DiMartino, J.F. *et al.* The AF10 leucine zipper is required for leukemic transformation of myeloid progenitors by MLL-AF10. *Blood* **99**, 3780-3785 (2002).

7. Tzelepis, K. *et al.* A CRISPR Dropout Screen Identifies Genetic Vulnerabilities and Therapeutic Targets in Acute Myeloid Leukemia. *Cell Rep* **17**, 1193-1205 (2016).

8. Cong, L. *et al.* Multiplex genome engineering using CRISPR/Cas systems. *Science* **339**, 819-823 (2013).

9. Labun, K. *et al.* CHOPCHOP v3: expanding the CRISPR web toolbox beyond genome editing. *Nucleic Acids Res* **47**, W171-W174 (2019).

10. Ray, D. *et al.* A compendium of RNA-binding motifs for decoding gene regulation. *Nature* **499**, 172-177 (2013).

11. Kozomara, A., Birgaoanu, M. & Griffiths-Jones, S. miRBase: from microRNA sequences to function. *Nucleic Acids Res* **47**, D155-D162 (2019).

12. Amaral, P.P., Clark, M.B., Gascoigne, D.K., Dinger, M.E. & Mattick, J.S. lncRNAdb: a reference database for long noncoding RNAs. *Nucleic Acids Res* **39**, D146-151 (2011).

13. Schneider, C.A., Rasband, W.S. & Eliceiri, K.W. NIH Image to ImageJ: 25 years of image analysis. *Nat Methods* **9**, 671-675 (2012).

14. Dobin, A. *et al.* STAR: ultrafast universal RNA-seq aligner. *Bioinformatics* **29**, 15-21 (2013).

15. Li, B. & Dewey, C.N. RSEM: accurate transcript quantification from RNA-Seq data with or

without a reference genome. *BMC Bioinformatics* **12**, 323 (2011).

16. Robinson, M.D., McCarthy, D.J. & Smyth, G.K. edgeR: a Bioconductor package for differential expression analysis of digital gene expression data. *Bioinformatics* **26**, 139-140 (2010).

17. Hao, Y. *et al.* Integrated analysis of multimodal single-cell data. *Cell* **184**, 3573-3587 e3529 (2021).

18. Li, Y. *et al.* Single-Cell Analysis of Neonatal HSC Ontogeny Reveals Gradual and Uncoordinated Transcriptional Reprogramming that Begins before Birth. *Cell Stem Cell* **27**, 732-747 e737 (2020).

19. Haferlach, T. *et al.* Clinical utility of microarray-based gene expression profiling in the diagnosis and subclassification of leukemia: report from the International Microarray Innovations in Leukemia Study Group. *J Clin Oncol* **28**, 2529-2537 (2010).

Gating strategy of FACS analysis in this study

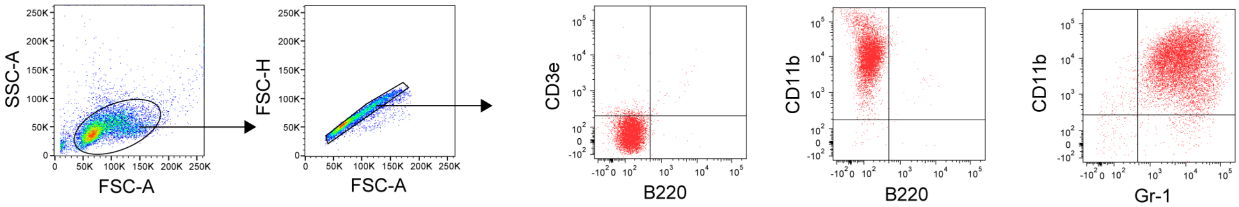

Uncropped scans of western blotting data in this supplementary information

Supplementary Figure 1c

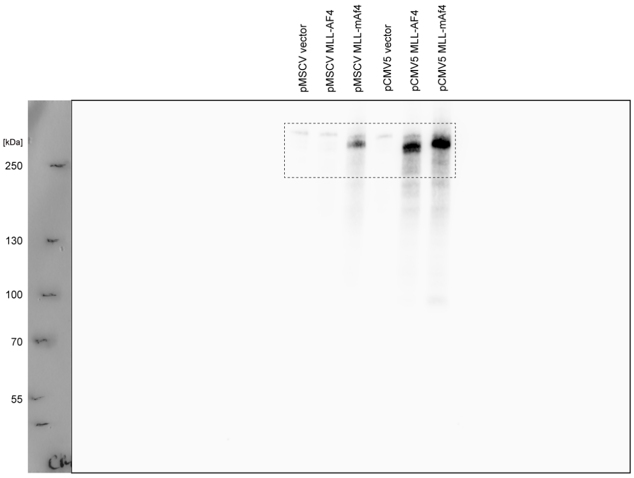

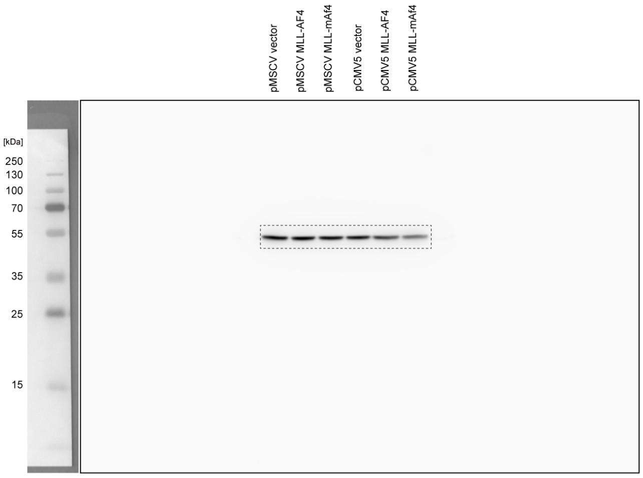

MLL Antibody (CST, #14689, 1/1000 dilution)

TUBB Antibody (Thermo, #PA5-25050, 0.5 μg/ml)

Protein ladder: PageRuler™ Plus Prestained Protein Ladder, 10 to 250 kDa (Thermo, #26620)

Supplementary Figure 2b

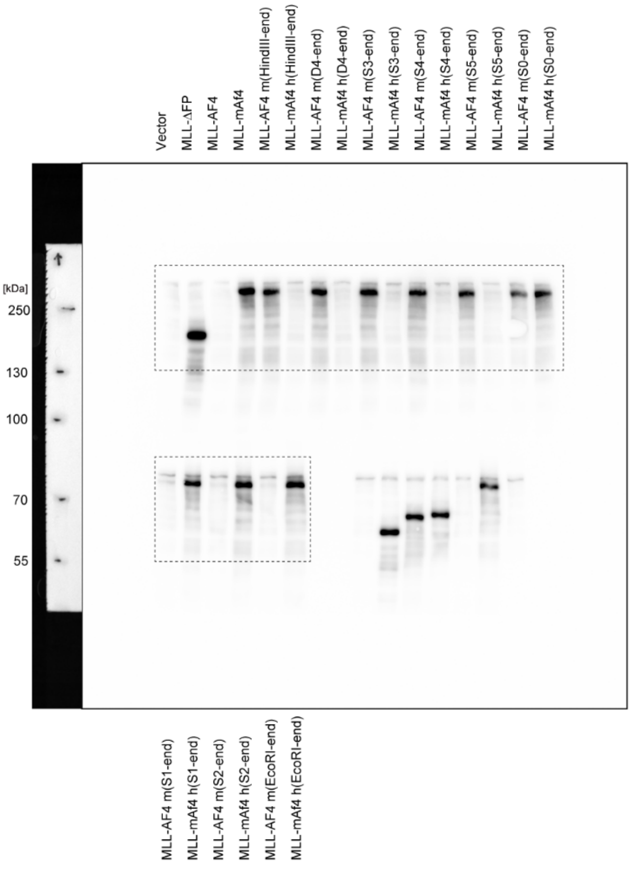

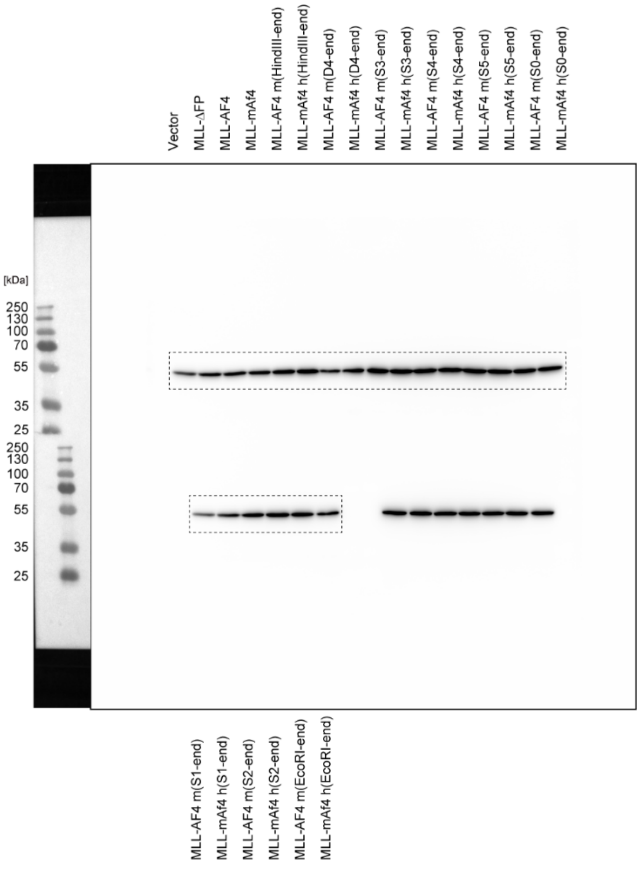

MLL Antibody (CST, #14689, 1/1000 dilution)

TUBB Antibody (Thermo, #PA5-25050, 0.5 μg/ml)

Protein ladder: PageRuler™ Plus Prestained Protein Ladder, 10 to 250 kDa (Thermo, #26620)

Supplementary Figure 2f

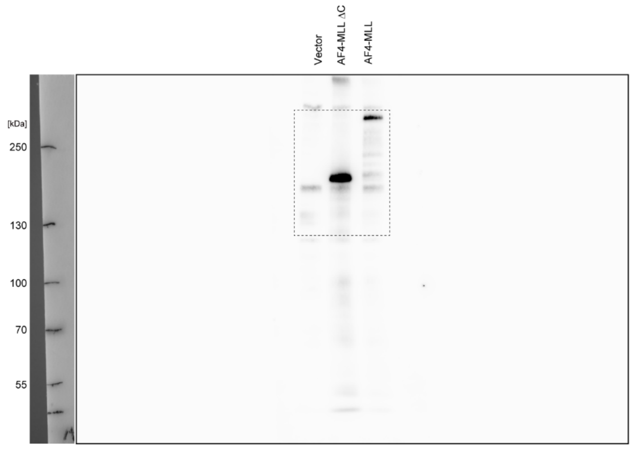

AF4 Antibody (Bethyl laboratories, #A302-344A, 1 μg/ml)

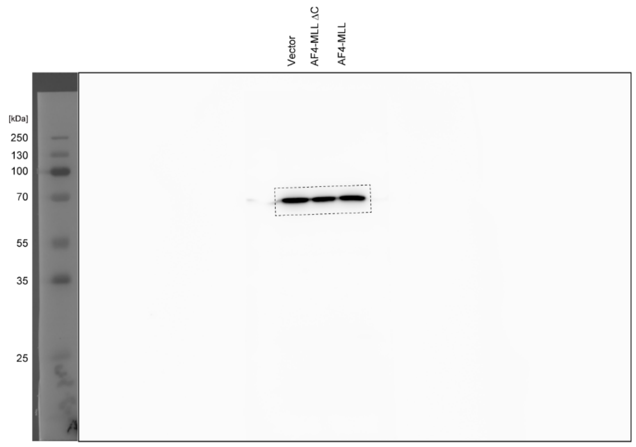

TUBB Antibody (Thermo, #PA5-25050, 0.5 μg/ml)

Protein ladder: PageRuler™ Plus Prestained Protein Ladder, 10 to 250 kDa (Thermo, #26620)

Supplementary Figure 4a

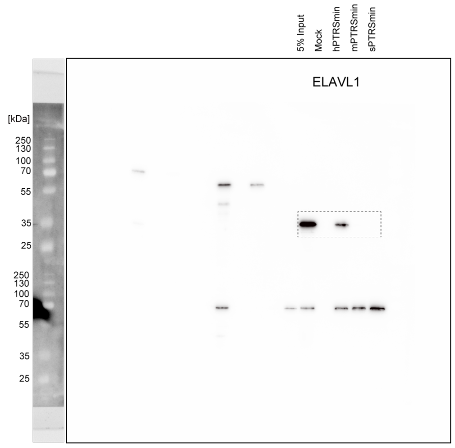

FLAG Antibody (Sigma, #F3165, 1 μg/ml)

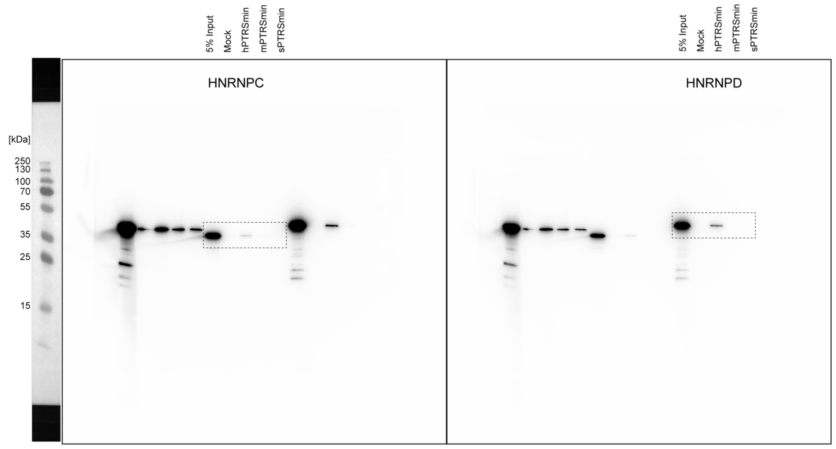

FLAG Antibody (Sigma, #F3165, 1 μg/ml)

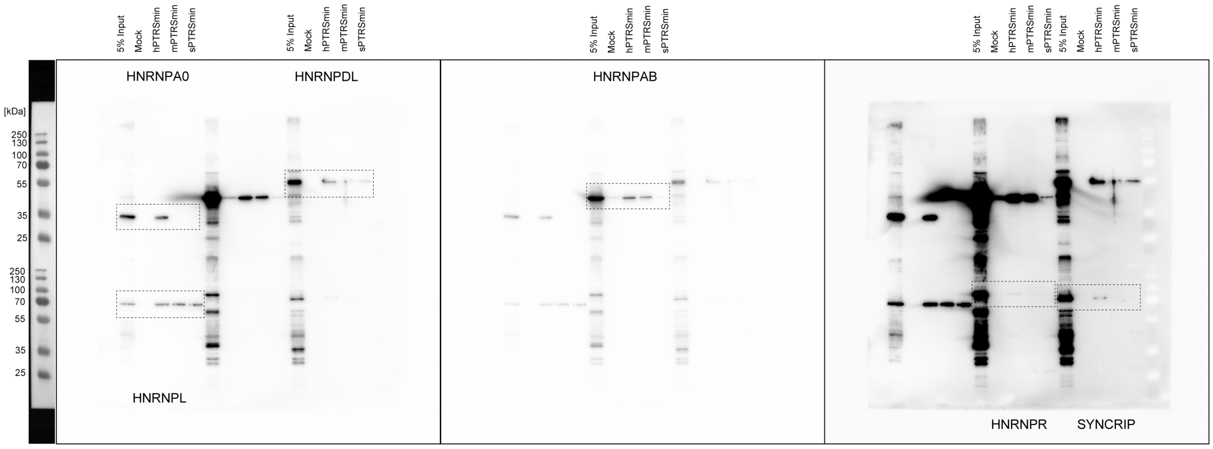

FLAG Antibody (Sigma, #F3165, 1 μg/ml)

Protein ladder: PageRuler™ Plus Prestained Protein Ladder, 10 to 250 kDa (Thermo, #26620)

Supplementary Figure 4e

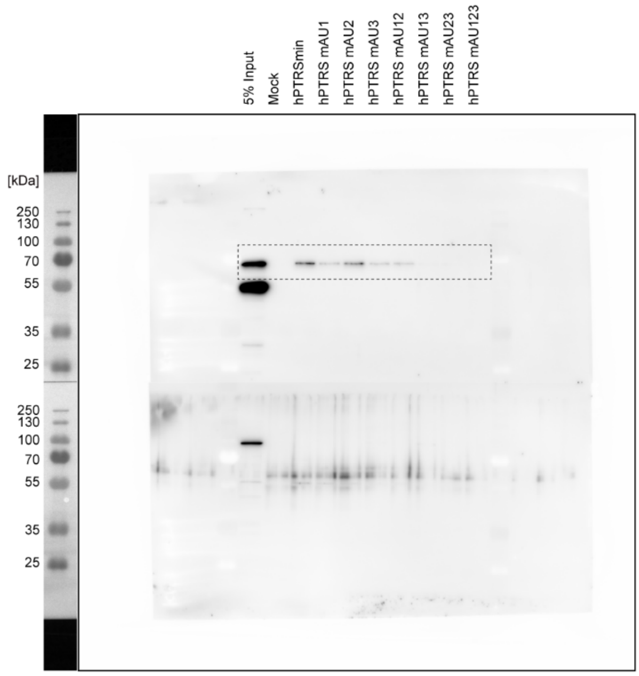

Upper : KHDRBS1 Antibody
(Bethyl laboratories, #A302-110A, 1 μg/ml)

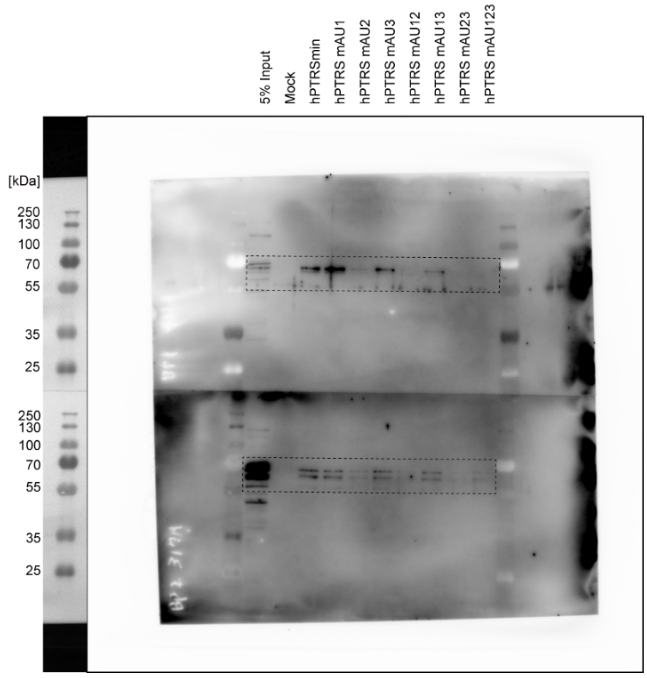

Upper : IGF2BP1 Antibody
(Bethyl laboratories, #A303-424A, 1 μg/ml)
Lower : IGF2BP2 Antibody
(Bethyl laboratories, #A303-317A, 1 μg/ml)

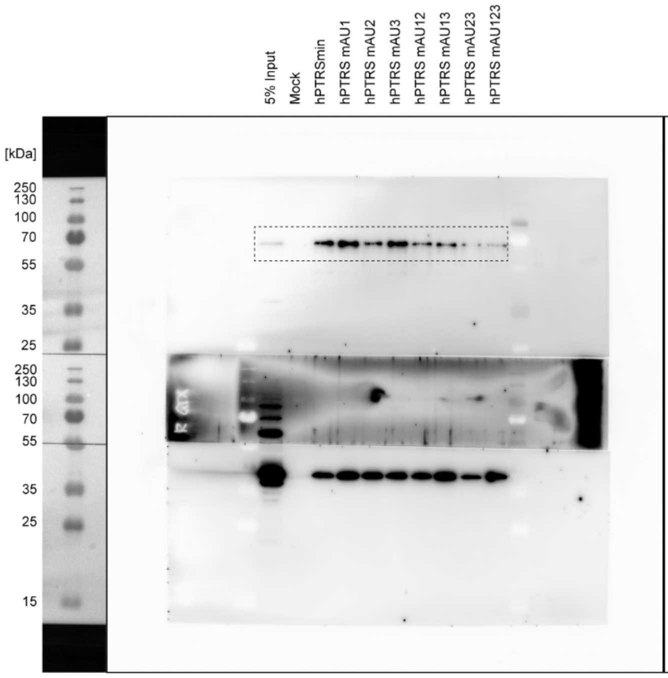

Top : IGF2BP3 Antibody
(Bethyl laboratories, #A303-426A, 1 μg/ml)

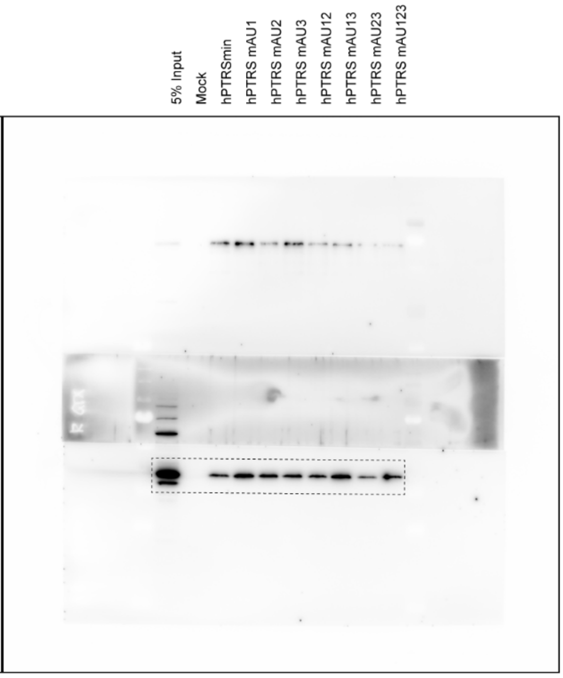

Bottom : HNRNPAB Antibody
(SantaCruz, #sc-376411, 1 μg/ml)

Supplementary Figure 4f

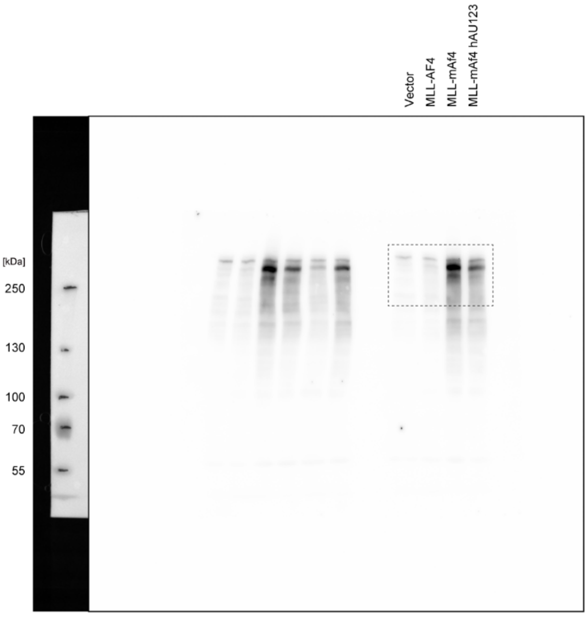

MLL Antibody (CST, #14689, 1/1000 dilution)

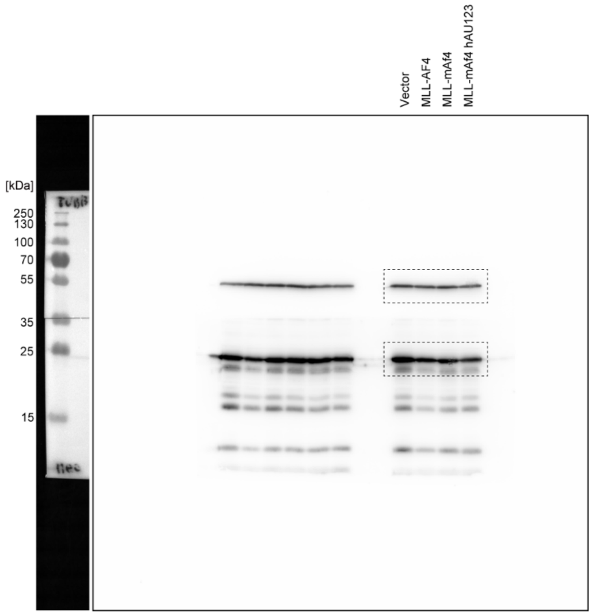

Upper : TUBB Antibody (Thermo, #PA5-25050, 0.5 μg/ml)
Lower : NPTII Antibody (Millipore, #06-747, 1 μg/ml)

Protein ladder: PageRuler™ Plus Prestained Protein Ladder, 10 to 250 kDa (Thermo, #26620)

Supplementary Figure 4i

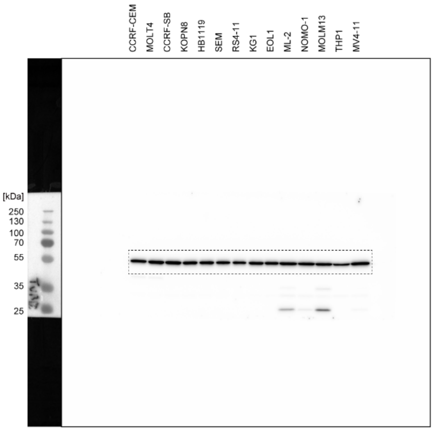

TUBB Antibody (Thermo, #PA5-25050, 0.5 μg/ml)

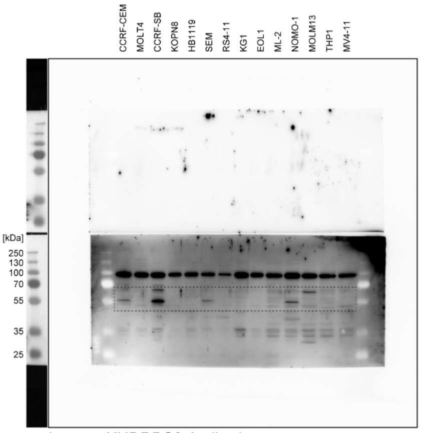

Lower : KHDRBS3 Antibody
(Bethyl laboratories, #A303-191A, 1 μg/ml)

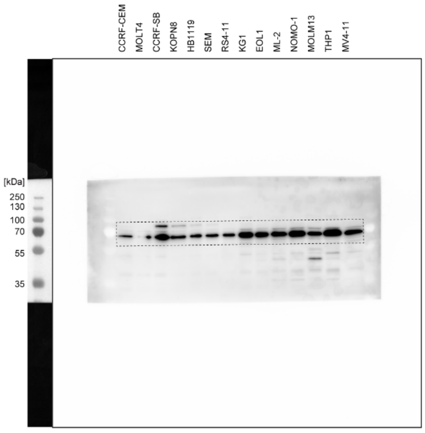

KHDRBS1 Antibody
(Bethyl laboratories, #A302-111A, 1 μg/ml)

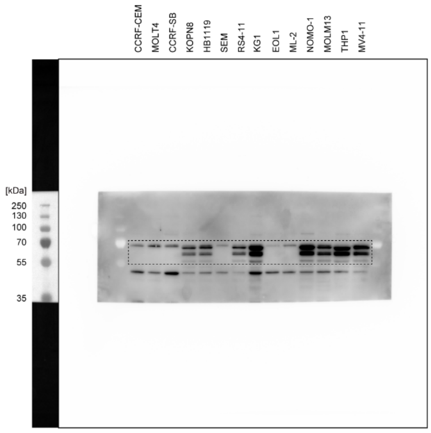

IGF2BP2 Antibody
(Bethyl laboratories, #A303-317A, 1 μg/ml)

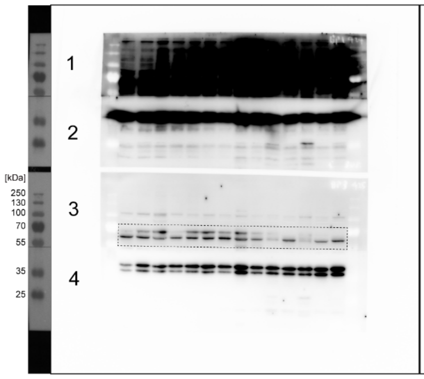

Third low : IGF2BP3 Antibody
(Bethyl laboratories, #A303-425A, 1 μg/ml)

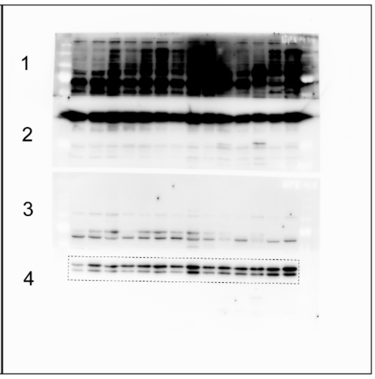

Fourth low : IGF2BP3 Antibody
(Bethyl laboratories, #A303-425A, 1 μg/ml)

Protein ladder: PageRuler™ Plus Prestained Protein Ladder, 10 to 250 kDa (Thermo, #26620)

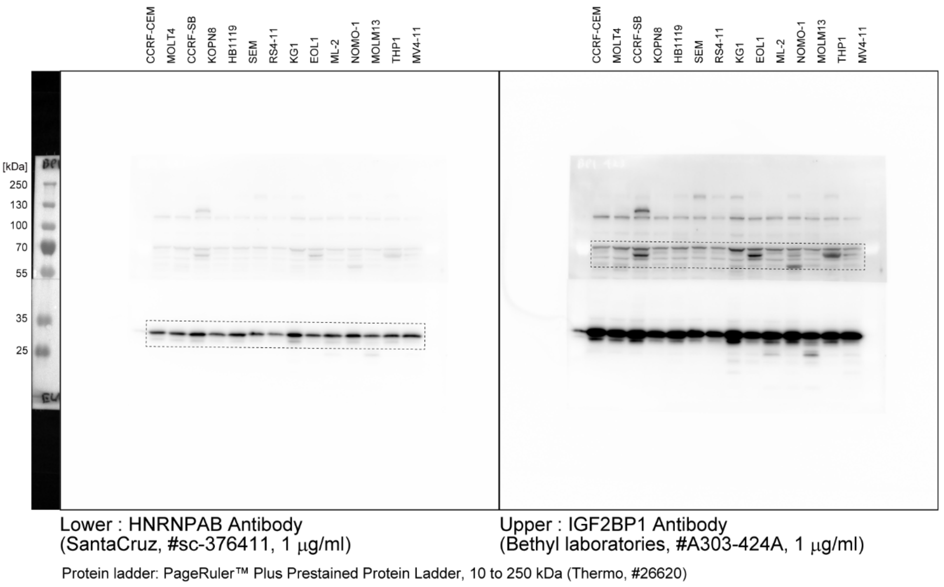

Supplementary Figure 5c

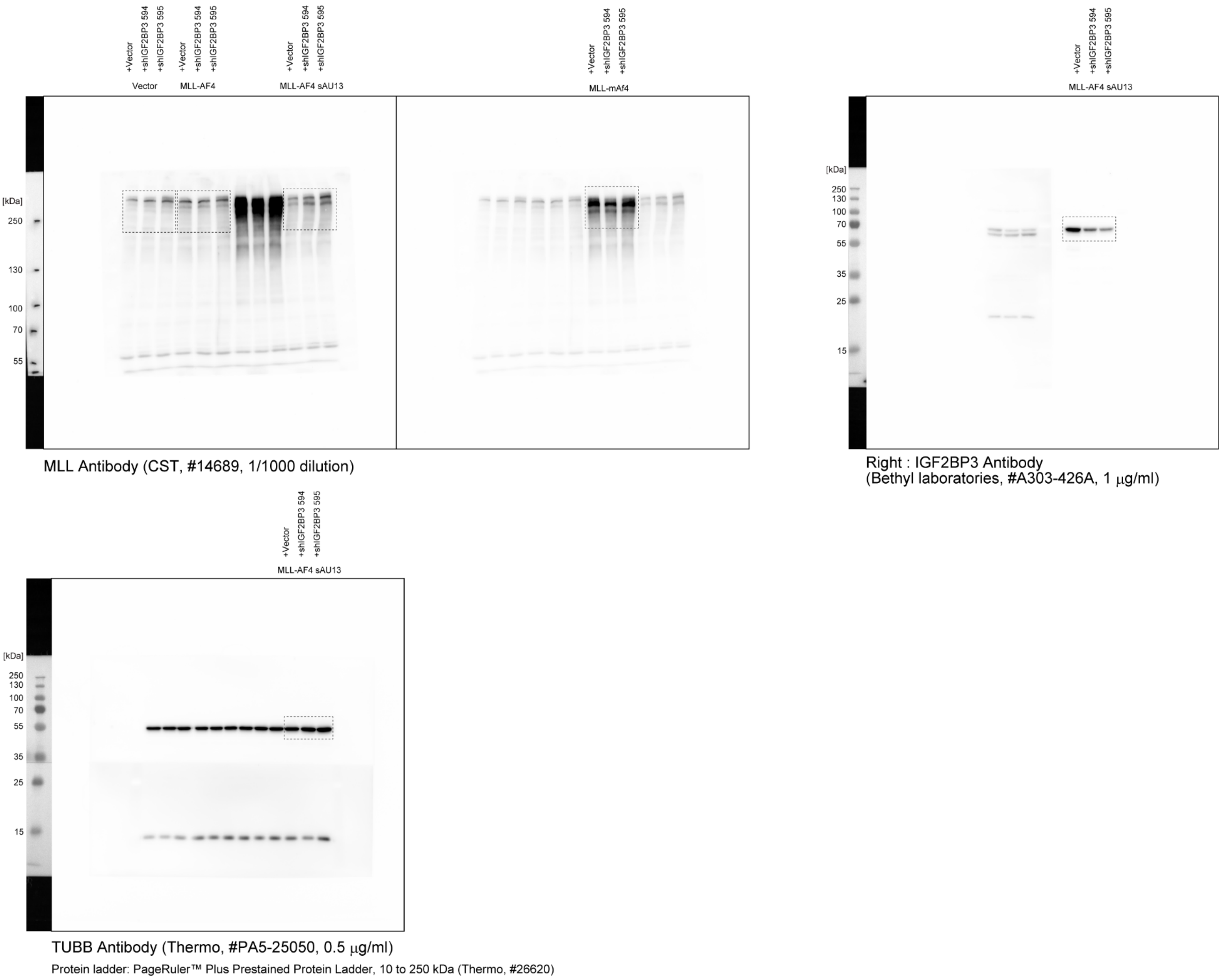

Supplementary Figure 6a

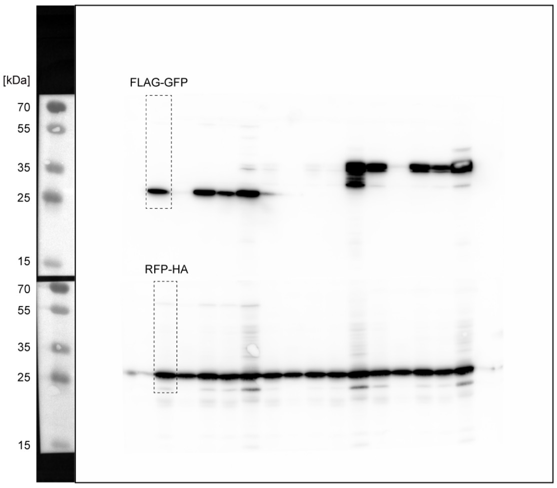

Upper : FLAG Antibody (Sigma, #F3165, 1 μg/ml)
Lower : HA Antibody (Roche, #11867423001, 1 μg/ml)

Protein ladder: PageRuler™ Plus Prestained Protein Ladder, 10 to 250 kDa (Thermo, #26620)

Supplementary Figure 6c

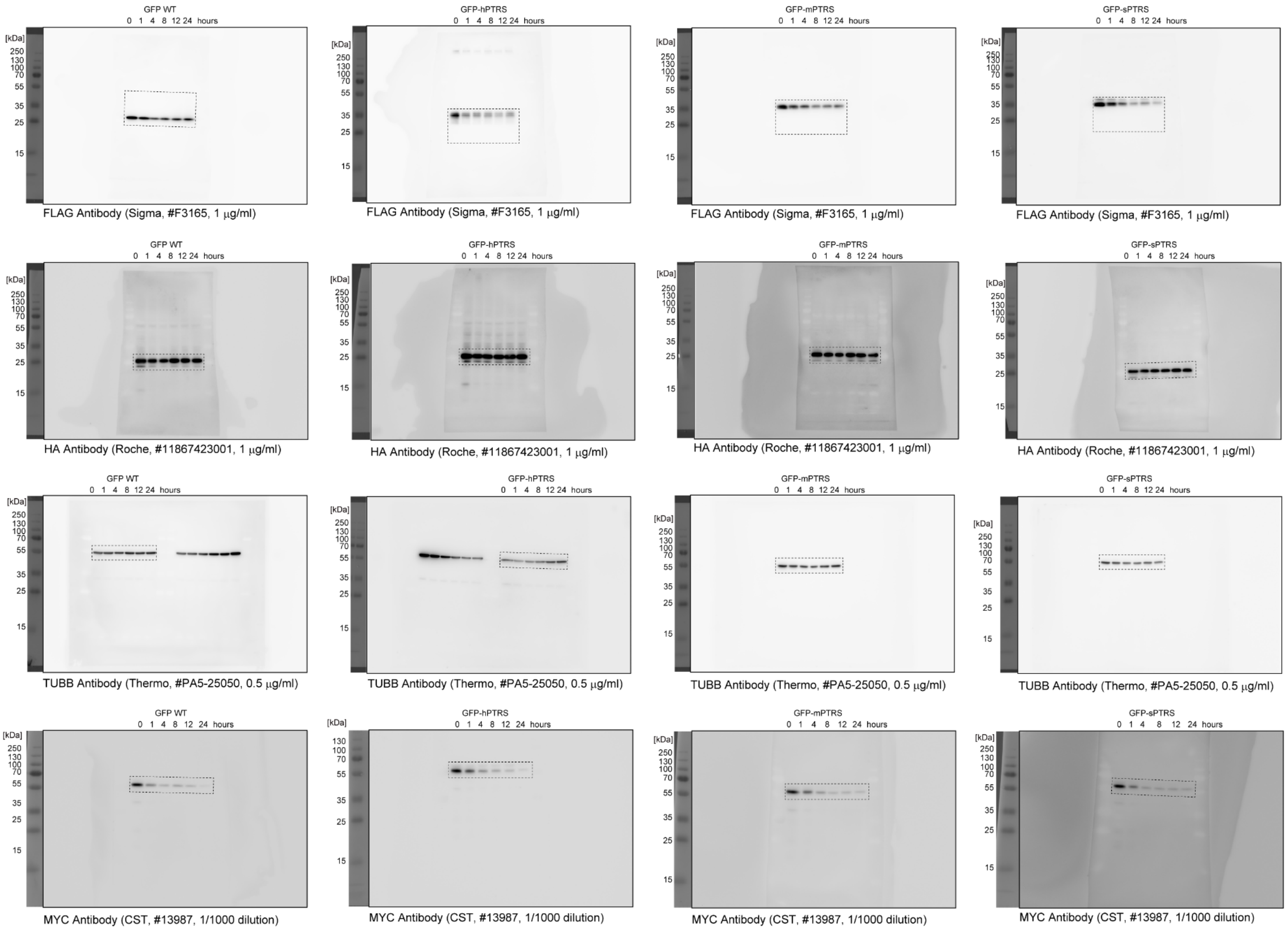

Protein ladder: PageRuler™ Plus Prestained Protein Ladder, 10 to 250 kDa (Thermo, #26620)

Supplementary Figure 6e

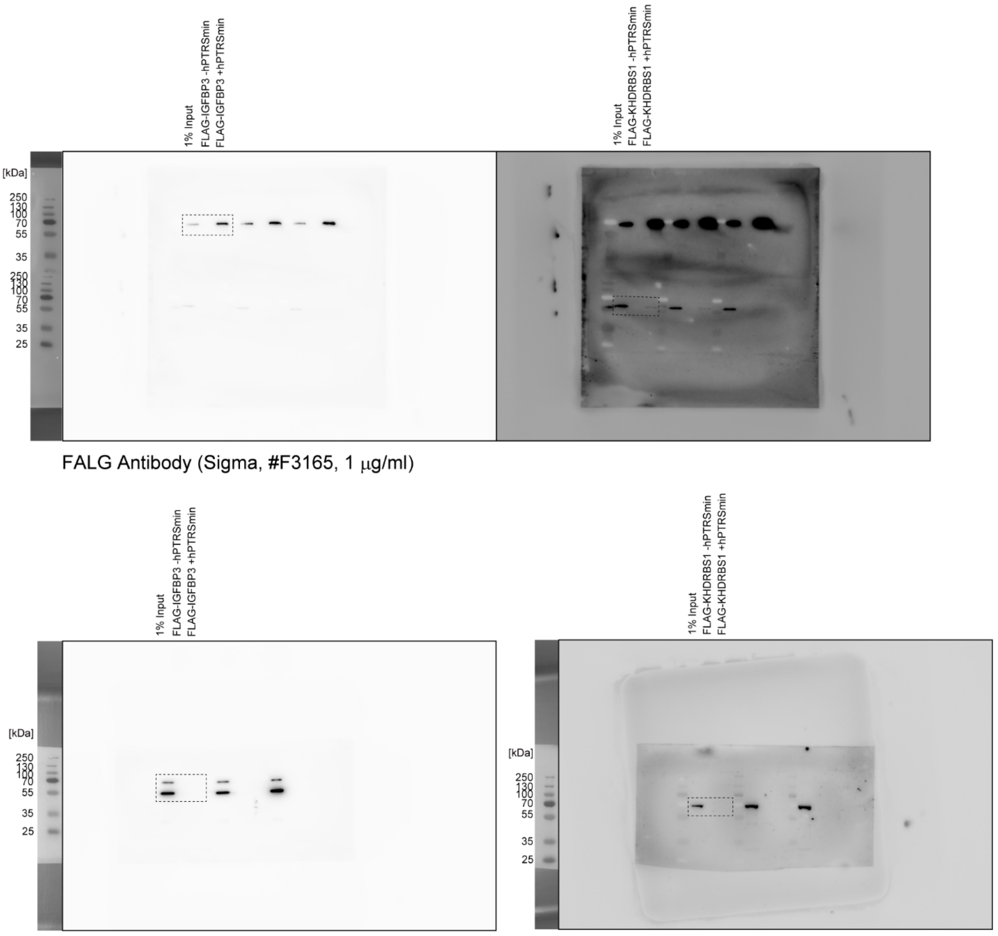

FALG Antibody (Sigma, #F3165, 1 μg/ml)

KHDRBS1 Antibody
(Bethyl laboratories, #A302-110A, 1 μg/ml)

IGF2BP3 Antibody
(Bethyl laboratories, #A303-426A, 1 μg/ml)

Protein ladder: PageRuler™ Plus Prestained Protein Ladder, 10 to 250 kDa (Thermo, #26620)
