# Supplementary material for: RNA-binding proteins of KHDRBS and IGF2BP families control the oncogenic activity of MLL-AF4
Source: Nat Commun. 2022 Nov 5;13:6688. doi: 10.1038/s41467-022-34558-1 (PMC9637093; doi:10.1038/s41467-022-34558-1)
Supplement: Supplementary file 3 — Reporting Summary [file 41467_2022_34558_MOESM3_ESM.pdf]

nature research

Corresponding author(s): Hiroshi Okuda and Akihiko Yokoyama

Last updated by author(s): Oct 24, 2022

Reporting Summary

Nature Research wishes to improve the reproducibility of the work that we publish. This form provides structure for consistency and transparency in reporting. For further information on Nature Research policies, see our Editorial Policies and the Editorial Policy Checklist.

Statistics

For all statistical analyses, confirm that the following items are present in the figure legend, table legend, main text, or Methods section.

n/a	Confirmed	
☐	☒	The exact sample size (n) for each experimental group/condition, given as a discrete number and unit of measurement
☐	☒	A statement on whether measurements were taken from distinct samples or whether the same sample was measured repeatedly
☐	☒	The statistical test(s) used AND whether they are one- or two-sided<br>*Only common tests should be described solely by name; describe more complex techniques in the Methods section.*
☒	☐	A description of all covariates tested
☒	☐	A description of any assumptions or corrections, such as tests of normality and adjustment for multiple comparisons
☐	☒	A full description of the statistical parameters including central tendency (e.g. means) or other basic estimates (e.g. regression coefficient) AND variation (e.g. standard deviation) or associated estimates of uncertainty (e.g. confidence intervals)
☐	☒	For null hypothesis testing, the test statistic (e.g. F, t, r) with confidence intervals, effect sizes, degrees of freedom and P value noted<br>*Give P values as exact values whenever suitable.*
☒	☐	For Bayesian analysis, information on the choice of priors and Markov chain Monte Carlo settings
☒	☐	For hierarchical and complex designs, identification of the appropriate level for tests and full reporting of outcomes
☒	☐	Estimates of effect sizes (e.g. Cohen's d, Pearson's r), indicating how they were calculated

Our web collection on statistics for biologists contains articles on many of the points above.

Software and code

Policy information about availability of computer code

Data collection	RT-qPCR data were collected with StepOnePlus or QuantStudio 3 Real time PCR systems (Thermo Fisher). FACS data were collected with FACSmelody Cell Sorter with FACSChorus software ver.1.3 or FACSCelesta flow cytometer with FACSDiva software ver.8.0.1.1 (BD Biosciences). Western blotting data were collected with FUSION FX imaging system (VILBER). All cell images were visualized using the BZ-X710 microscope (KEYENCE). Mass spectrometry data were collected using LTQ Orbitrap ELITE ETD mass spectrometer with Xcalibur software (Thermo).
Data analysis	All statistical data were analyzed with Prism 9.4.1 software (GraphPad software). All FACS data were analyzed with FlowJo software (v.10.6.1).

For manuscripts utilizing custom algorithms or software that are central to the research but not yet described in published literature, software must be made available to editors and reviewers. We strongly encourage code deposition in a community repository (e.g. GitHub). See the Nature Research guidelines for submitting code & software for further information.

Data

Policy information about availability of data

All manuscripts must include a data availability statement. This statement should provide the following information, where applicable:

- Accession codes, unique identifiers, or web links for publicly available datasets
- A list of figures that have associated raw data
- A description of any restrictions on data availability

The mass spectrometry data used in Figure 4b and Figure S6c are deposited in the JPOST repository
https://repository.jpostdb.org
JPOST ID: JPST001132
The NGS data were deposited in Gene Expression Omnibus (GSE201503).

Field-specific reporting

Please select the one below that is the best fit for your research. If you are not sure, read the appropriate sections before making your selection.

☒ Life sciences ☐ Behavioural & social sciences ☐ Ecological, evolutionary & environmental sciences

For a reference copy of the document with all sections, see nature.com/documents/nr-reporting-summary-flat.pdf

Life sciences study design

All studies must disclose on these points even when the disclosure is negative.

Sample size	No statistical method was used to predetermine the sample size. The chosen sample size are based on the numbers used for previous publications.
Data exclusions	Figure 2 was presented with the data from which some leukemic mice were excluded for the reasons described in the Supplementary figure 3.
Replication	All experiments were repeated independently as indicated in each Figure Legend. Animal experiments were repeated thrice.
Randomization	Randomization was not applied to the experiments described in the study.
Blinding	Investigators were not blinded to the sample identities during data collection.

Reporting for specific materials, systems and methods

We require information from authors about some types of materials, experimental systems and methods used in many studies. Here, indicate whether each material, system or method listed is relevant to your study. If you are not sure if a list item applies to your research, read the appropriate section before selecting a response.

Materials & experimental systems

n/a	Involved in the study
☐	☒ Antibodies
☐	☒ Eukaryotic cell lines
☒	☐ Palaeontology and archaeology
☐	☒ Animals and other organisms
☒	☐ Human research participants
☒	☐ Clinical data
☒	☐ Dual use research of concern

Methods

n/a	Involved in the study
☒	☐ ChIP-seq
☐	☒ Flow cytometry
☒	☐ MRI-based neuroimaging

Antibodies

Antibodies used

Anti-FLAG M2 (Sigma, clone M2, F3165, 1 µg/ml)
Anti-FLAG (Sigma, F7425, 1 µg/ml)
Anti-HA (Roche, clone 3F10, 11867423001, 1 µg/ml)
Anti-MLL (Cell Signalling Technology, clone D2M7U, 14689, 1/1000 dilution)
Anti-TUBB2C (Invitrogen, PA5-25050, 0.5 µg/ml)
Anti-neomycin phosphotransferase II (Merck-Millipore, 06-747, 1 µg/ml)
Anti-KHDRBS1 (Bethyl Laboratories, A302-110A, 1 µg/ml)
Anti-KHDRBS1 (Bethyl Laboratories, A302-111A, 1 µg/ml)
Anti-KHDRBS3 (Bethyl Laboratories, A303-192A, 1 µg/ml)
Anti-IGF2BP1 (Bethyl Laboratories, A303-423A, 1 µg/ml)
Anti-IGF2BP1 (Bethyl Laboratories, A303-424A, 1 µg/ml)
Anti-IGF2BP2 (Bethyl Laboratories, A303-317A, 1 µg/ml)
Anti-IGF2BP3 (Bethyl Laboratories, A303-425A, 1 µg/ml)
Anti-IGF2BP3 (Bethyl Laboratories, A303-426A, 1 µg/ml)
Anti-HNRNPAB (Santa Cruz Biotechnology, clone G10, sc-376411, 1 µg/ml)
Anti-RPL5 (Cell Signalling Technology, 51345, 1/1000 dilution)
Anti-RPL7a (Cell Signalling Technology, 2415, 1/1000 dilution)
Anti-RPL7 (Bethyl Laboratories, A300-741A, 1 µg/ml)
Anti-RPS14 (Bethyl Laboratories, A304-031A, 1 µg/ml)
Anti-AF4 (Bethyl Laboratories, A302-344A, 1 µg/ml)
Anti-MYC (Cell Signalling Technology, 13987, 1/1000)
Goat anti-Mouse IgG (H+L) Secondary Antibody, HRP (Thermo, 31430, 1/10000 dilution)
Goat anti-Rabbit IgG (H+L) Secondary Antibody, HRP (Thermo, 31460, 1/10000 dilution)
Anti-human/mouse CD45R(B220) (clone RA3-6B2)-FITC (eBioscience, 11-0452-82, 1/100 dilution)

Anti-human/mouse CD45R(B220) (clone RA3-6B2)-PE (BioLegend, 103208, 1/500 dilution)
Anti-mouse CD3e (clone 145-2C11)-APC (eBioscience, 17-0031-82, 1/100 dilution)
Anti-mouse CD3e (clone 145-2C11)-APC (BioLegend, 100312, 1/200 dilution)
Anti-mouse CD11b (clone M1/70)-PE-Cyanine7 (eBioscience, 25-0112-82,1/200 dilution)
Anti-mouse CD11b (clone M1/70)-Brilliant Violet 510 (BioLegend, 101263, 1/500 dilution)
Anti-mouse Gr1 (clone RB6-8C5)-PE-Cyanine7 (eBioscience, 25-5931-82, 1/200 dilution)
Anti-mouse Gr1 (clone RB6-8C5)-Brilliant Violet 421 (BD Biosciences, 562709, 1/500 dilution)

Validation

All antibodies were commercially available and validate by manufactures/suppliers.
The validation information is available the web sites listed below.
Anti-FLAG M2 (https://www.sigmaaldrich.com/catalog/product/sigma/f3165)
Anti-FLAG (https://www.sigmaaldrich.com/catalog/product/sigma/f7425)
Anti-HA (https://www.sigmaaldrich.com/catalog/product/roche/roahaha)
Anti-MLL (https://www.cellsignal.com/products/primary-antibodies/mll1-d2m7u-rabbit-mab-amino-terminal-antigen/14689)
Anti-TUBB2C (https://www.thermofisher.com/antibody/product/Tubulin-beta-2C-Antibody-Polyclonal/PA5-25050)
Anti-neomycin phosphotransferase II (https://www.emdmillipore.com/US/en/product/Anti-Neomycin-Phosphotransferase-II-Antibody,MM_NF-06-747)
Anti-KHDRBS1 (https://www.bethyl.com/product/A302-110A)
Anti-KHDRBS1 (https://www.bethyl.com/product/A302-111A)
Anti-KHDRBS3 (https://www.bethyl.com/product/A303-192A)
Anti-IGF2BP1 (https://www.bethyl.com/product/A303-423A)
Anti-IGF2BP1 (https://www.bethyl.com/product/A303-424A)
Anti-IGF2BP2 (https://www.bethyl.com/product/A303-317A)
Anti-IGF2BP3 (https://www.bethyl.com/product/A303-425A)
Anti-IGF2BP3 (https://www.bethyl.com/product/A303-426A)
Anti-HNRNPAB (https://www.scbt.com/p/hnrnp-a-b-antibody-g-10/)
Anti-RPL5 (https://www.cellsignal.com/products/primary-antibodies/rpl5-d5q5x-rabbit-mab/51345)
Anti-RPL7a (https://www.cellsignal.com/products/primary-antibodies/ribosomal-protein-l7a-e109-antibody/2415)
Anti-RPL7 (https://www.bethyl.com/product/A300-741A)
Anti-RPS14 (https://www.bethyl.com/product/A304-031A)
Anti-AF4 (https://www.bethyl.com/product/A302-344A)
Anti-MYC (https://www.cellsignal.com/products/primary-antibodies/c-myc-n-myc-d3n8f-rabbit-mab/13987)
Anti-Mouse IgG-HRP (https://www.thermofisher.com/antibody/product/Goat-anti-Mouse-IgG-H-L-Secondary-Antibody-Polyclonal/31430)
Anti-Rabbit IgG-HRP (https://www.thermofisher.com/antibody/product/Goat-anti-Rabbit-IgG-H-L-Secondary-Antibody-Polyclonal/31460)
Anti-CD45R-FITC (https://www.thermofisher.com/antibody/product/CD45R-B220-Antibody-clone-RA3-6B2-Monoclonal/11-0452-82)
Anti-CD45R-PE (https://www.biolegend.com/en-us/products/pe-anti-mouse-human-cd45r-b220-antibody-447)
Anti-CD3e-APC (https://www.thermofisher.com/(antibody/product/CD3e-Antibody-clone-145-2C11-Monoclonal/17-0031-82)
Anti-CD3e-APC (https://www.biolegend.com/en-us/products/apc-anti-mouse-cd3epsilon-antibody-21)
Anti-CD11b-PE-Cy7 (https://www.thermofisher.com/antibody/product/CD11b-Antibody-clone-M1-70-Monoclonal/25-0112-82)
Anti-CD11b-BV510 (https://www.biolegend.com/en-us/products/brilliant-violet-510-anti-mouse-human-cd11b-antibody-7993)
Anti-Gr1-PE-Cy7 (https://www.thermofisher.com/antibody/product/Ly-6G-Ly-6C-Antibody-clone-RB6-8C5-Monoclonal/17-5931-82)
Anti-Gr1-BV421 (https://www.bdbiosciences.com/en-us/products/reagents/flow-cytometry-reagents/research-reagents/single-color-antibodies-ruo/bv421-rat-anti-mouse-ly-6g-and-ly-6c.562709)

Eukaryotic cell lines

Policy information about cell lines

Cell line source(s)

293T (Human, ATCC, CRL-3216)
PLAT-E (Human, from T. Kitamura lab, see ref.40)
293TN (Human, System Bioscience, LV900A-1)
CCRF-CEM (Human, JCRB Cell Bank, JCRB0033)
MOLT-4 (Human, JCRB Cell Bank, JCRB9031)
CCRF-SB (Human, JCRB Cell Bank, JCRB0032)
KOPN-8 (Human, DSMZ, ACC552)
HB1119 (Human, from M.L. Cleary lab, see ref.39)
SEM (Human, DSMZ, ACC546)
RS4;11 (Human, ATCC, CRL-1873)
KG-1 (Human, JCRB Cell Bank, JCRB0065)
EOL-1 (Human, from M.L. Cleary lab)
ML-2 (Human, DSMZ, ACC15)
NOMO-1 (Human, JCRB Cell Bank, IFO50474)
MOLM-13 (Human, DSMZ, ACC554)
THP-1 (Human, ATCC, TIB-202)
MV4-11 (Human, ATCC, CRL-9591)
MS-5 (Mouse, DSMZ, ACC441)
MS-5-neo (Mouse, This study)
Ba/F3 (Mouse, RIKEN BRC)
MEF clone C2-20 (Mouse, our stock, see ref.7)

Authentication

All the cell lines commercially available are authenticated by the vendors at the purchase. HB1119 cells were authenticated by western blotting and RNA-seq analyses. EOL-1 cells were autheticated by JCRB cell bank. PLAT-E cells, a derivative of 293

	cells, were authenticated as 293 cells by JCRB cell bank and as PLAT-E cells by their virus production and resistance to antibiotics. The MEF clone C2-20 was established in-house using standard procedure and analyzed by RNA-seq.
Mycoplasma contamination	The commercially available cell lines were tested for mycoplasma contamination by the vendors. Cells that underwent long-term culture or those that were provided from the non-commercial sources were periodically tested negative for mycoplasma infection by using LONZA Mycoalert mycoplasma detection kit.
Commonly misidentified lines (See ICLAC register)	No commonly misidentified cell lines were used.

Animals and other organisms

Policy information about studies involving animals; ARRIVE guidelines recommended for reporting animal research

Laboratory animals	Female C57BL/6JJcl mice were obtained from CLEA Japan inc.. Five-week-old female C57BL/6JJcl mice were used for bone marrow extraction and seven-week-old mice were used for leukemogenesis assay. Mice were allowed free access to food and water and were maintained at room temperature (about 25C) with constant humidity (about 50%) on a 12-hours light/dark cycle.
Wild animals	No wild animals were used in this study.
Field-collected samples	No filed-collected samples were used in this study.
Ethics oversight	The mouse work was performed under the protocol which was approved by the National Cancer Center Institutional Animal Care and Use Committee and Institutional Review Board of Yokohama City University.

Note that full information on the approval of the study protocol must also be provided in the manuscript.

Flow Cytometry

Plots

Confirm that:

- [x] The axis labels state the marker and fluorochrome used (e.g. CD4-FITC).
- [x] The axis scales are clearly visible. Include numbers along axes only for bottom left plot of group (a 'group' is an analysis of identical markers).
- [ ] All plots are contour plots with outliers or pseudocolor plots.
- [x] A numerical value for number of cells or percentage (with statistics) is provided.

Methodology

Sample preparation	Bone marrow cells were harvested from the femurs and tibiae of leukemic mice. The red blood cells were removed via treatment with ACK lysis buffer (150 mM NH4Cl, 10 mM KHCO3, 0.1 mM EDTA), and the cells were then stained with antibodies in PBS with 3% FBS.
Instrument	Cells were analyzed using the FACSMelody cell sorter and FACSCelesta flow cytometer (BD Bioscience).
Software	The data were collected using FACSChorus and FACSDiva software (BD Bioscience) and analyzed using FlowJo software.
Cell population abundance	No sorting was performed.
Gating strategy	Cells were first gated on their FSC-A/SSC-A profile. Doublets were removed by FSC-A/FSC-H. Representative gating strategies are shown within the figures.

- [x] Tick this box to confirm that a figure exemplifying the gating strategy is provided in the Supplementary Information.
